# A time-resolved interaction analysis of Bem1 reconstructs the flow of Cdc42 during polar growth

**DOI:** 10.26508/lsa.202000813

**Published:** 2020-07-31

**Authors:** Sören Grinhagens, Alexander Dünkler, Yehui Wu, Lucia Rieger, Philipp Brenner, Thomas Gronemeyer, Medhanie A Mulaw, Nils Johnsson

**Affiliations:** 1 Department of Biology, Institute of Molecular Genetics and Cell Biology, Ulm University, Ulm, Germany; 2 Comprehensive Cancer Center Ulm, Institute of Experimental Cancer Research, Ulm University, Ulm, Germany

## Abstract

Cdc42 organizes cellular polarity and directs the formation of cellular structures in many organisms. By locating Cdc24, the source of active Cdc42, to the growing front of the yeast cell, the scaffold protein Bem1, is instrumental in shaping the cellular gradient of Cdc42. This gradient instructs bud formation, bud growth, or cytokinesis through the actions of a diverse set of effector proteins. To address how Bem1 participates in these transformations, we systematically tracked its protein interactions during one cell cycle to define the ensemble of Bem1 interaction states for each cell cycle stage. Mutants of Bem1 that interact with only a discrete subset of the interaction partners allowed to assign specific functions to different interaction states and identified the determinants for their cellular distributions. The analysis characterizes Bem1 as a cell cycle–specific shuttle that distributes active Cdc42 from its source to its effectors. It further suggests that Bem1 might convert the PAKs Cla4 and Ste20 into their active conformations.

## Abstract (precis)

A temporal dissection correlates changes in composition and architecture of the interaction network of Bem1 with changes in cellular morphology and the activities of its binding partners.

## Introduction

Bud formation, growth, and cell separation are the visible consequences of polar cell growth in the budding yeast (Bi & Park, 2012; Howell & Lew, 2012). Interactions between the involved cell polarity proteins might act as switches to drive these morphological alterations. Accordingly, changes in the composition and structure of the protein interaction network should correlate with the different phases of cell growth.

Yeast cells initiate bud formation at a predetermined site, expand the bud preferentially at its tip, switch in large buds to an isotropic growth, and finally reorient the growth axis during mitosis and cell separation (Howell & Lew, 2012). The Rho-like GTPase Cdc42 influences local cell expansion in all cell cycle phases by binding in its active, GTP-bound state to different effector proteins (Chiou et al, 2017). Cdc42_GTP_ instructs the organization of the septin- and actin cytoskeleton, the spatial organization of exocytosis, mating, osmolarity sensing, and mitotic exit (Pruyne et al, 2004; Bi & Park, 2012). Cdc24, the guanine-nucleotide-exchange factor (GEF) for Cdc42, and a variety of (GAPs) GTPase-activating protein adjust the concentration of Cdc42_GTP_ at the cortex (Smith et al, 2002). The concentration of Cdc42_GTP_ changes over the cell cycle and peaks at the G1/S and at anaphase (Atkins et al, 2013).

Bem1 is the central scaffold for proteins that organize polarized growth in yeast (Chenevert et al, 1992; Peterson et al, 1994; Bender et al, 1996; Matsui et al, 1996). Bem1 binds Cdc24, Cdc42_GTP_, and several Cdc42_GTP_ effector proteins (Bose et al, 2001; Irazoqui et al, 2003). The protein is part of the polarity cap during bud growth, cell separation, cell mating, and fusion and assists Cdc42 in the pheromone response-, the filamentous growth-, and the high osmolarity MAPK pathways (Lyons et al, 1996; Leberer et al, 1997; Winters & Pryciak, 2005; Tanaka et al, 2014).

During G1, Bem1 plays a key role in polarity establishment by forming a stable zone of Cdc42_GTP_ at the cell cortex. Physically connecting Ccd24 to Cdc42_GTP_, Bem1 organizes a positive feedback where Cdc42_GTP_ attracts further Cdc24 to activate even more Cdc42 (Irazoqui et al, 2003; Kozubowski et al, 2008; Woods et al, 2015; Witte et al, 2017).

Bem1 consists of two N-terminally located (SH3) SRC homology 3 domains (SH3_a_ and SH3_b_), a lipid-binding (PX) phox homology domain, and a C-terminal (PB1) Phox and Bem1 domain (PB1_Bem1_) (Bender et al, 1996; Matsui et al, 1996). SH3_b_ interacts with well-characterized PxxP motifs in the p21 activated kinase (PAKs) Cla4 and Ste20, and the polarity proteins Boi1 and Boi2 (Bender et al, 1996; Bose et al, 2001; Winters & Pryciak, 2005; Gorelik & Davidson, 2012). SH3_b_ harbors a C-terminal extension (CI) that binds Cdc42_GTP_ (Yamaguchi et al, 2007; Takaku et al, 2010). PB1_Bem1_ binds the C-terminal PB1 domain of Cdc24 with high affinity and localizes Cdc24 to sites of polar growth during all cell cycle stages (Butty et al, 2002; Woods et al, 2015; Witte et al, 2017). The mechanisms of Bem1’s precisely regulated cellular distribution are, however, not fully understood (Woods et al, 2015; Meca et al, 2019).

Linking Cdc42 to Cdc24 might not suffice to explain the many functions of Bem1 during the other phases of the cell cycle (Atkins et al, 2008; Kozubowski et al, 2008; Li & Wedlich-Soldner, 2009). Instead, Bem1 was also shown to modestly stimulate Cdc24’s GEF activity (Smith et al, 2013; Rapali et al, 2017). By simultaneously binding to Cla4/Ste20, active Cdc42, and Cdc24, Bem1 might also induce a negative feedback to tone down the activity of Cdc24 during later stages of the cell cycle (Gulli et al, 2000; Kozubowski et al, 2008; Kuo et al, 2014; Rapali et al, 2017).

Here, we probe the interaction network of Bem1 throughout polar growth and cytokinesis to correlate changes in composition and architecture of the network with changes in cellular morphology and the activities of its binding partners.

## Results

### A protein interaction map of Bem1

**Figure 1.**
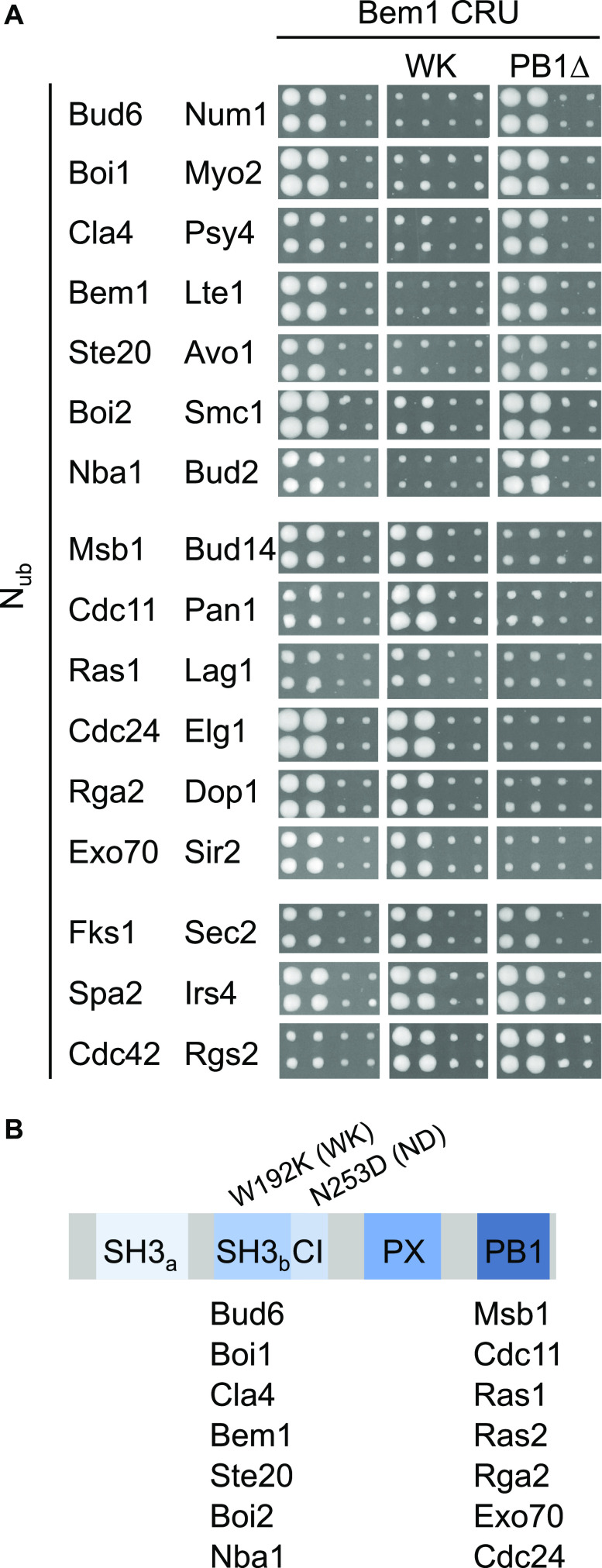
Interaction partners of Bem1. **(A)** Yeast cells carrying Bem1CRU or either of its two mutants Bem1_WK_- and Bem1_PB1∆_CRU were independently mated four times with N_ub_ fusion expressing strains. Interaction is indicated by growth of the four matings on SD medium containing 5-fluoro-orotic acid. Shown are the cut outs of the quadruplets expressing the N_ub_ fusion of the interacting protein on the left, next to a fusion that does not interact. **(B)** The domains of Bem1 and the positions of the residue exchanges of the *bem1*-alleles used in this work. The domain-specific interaction partners of (A) are listed below the respective domains.

We searched for binding partners of Bem1 by performing a systematic split-ubiquitin (Split-Ub) screen of Bem1-C_ub_-RUra3 (Bem1CRU) against 548 N_ub_ fusion proteins known or suspected to be involved in different aspects of polarized growth in yeast (see the Materials and Methods section) (Johnsson & Varshavsky, 1994; Hruby et al, 2011). The screen identified besides known binding partners, Bud6, Msb1, Ras1, Ras2, Rga2, Nba1, Spa2, Cdc11, Fks1, and Bem1 as novel interaction partners of Bem1 (Fig 1A). We repeated the screen with mutants of Bem1 that either carried the well-characterized W192K exchange in SH3_b_ (Bem1_WK_CRU) or lacked the C-terminal PB1 domain, and thus, the binding site to Cdc24 (Bem1_∆PB1_CRU) (Fig 1A and B). The comparison with Bem1CRU fusion revealed that Bem1_WK_ lost its interactions to Boi1, Ste20, Cla4, Bud6, Nba1, and Bem1 and showed a strongly reduced binding to Boi2 but retained its interactions with Exo70, Cdc24, Cdc42, Cdc11, Rga2, Msb1, Ras2, and Ras1 (Fig 1A and B). Deleting the PB1 domain in Bem1_PB1∆_CRU removed or strongly reduced the interactions of Bem1 to N_ub_-Cdc24, -Cdc11, -Rga2, -Msb1, -Ras1, -Ras2, and -Exo70 (Fig 1A and B). Neither mutation visibly affected the interaction of Bem1 with Fks1, Cdc42, or Spa2 (Fig 1A).

### Dissection of the Bem1 interaction network

**Figure 2.**
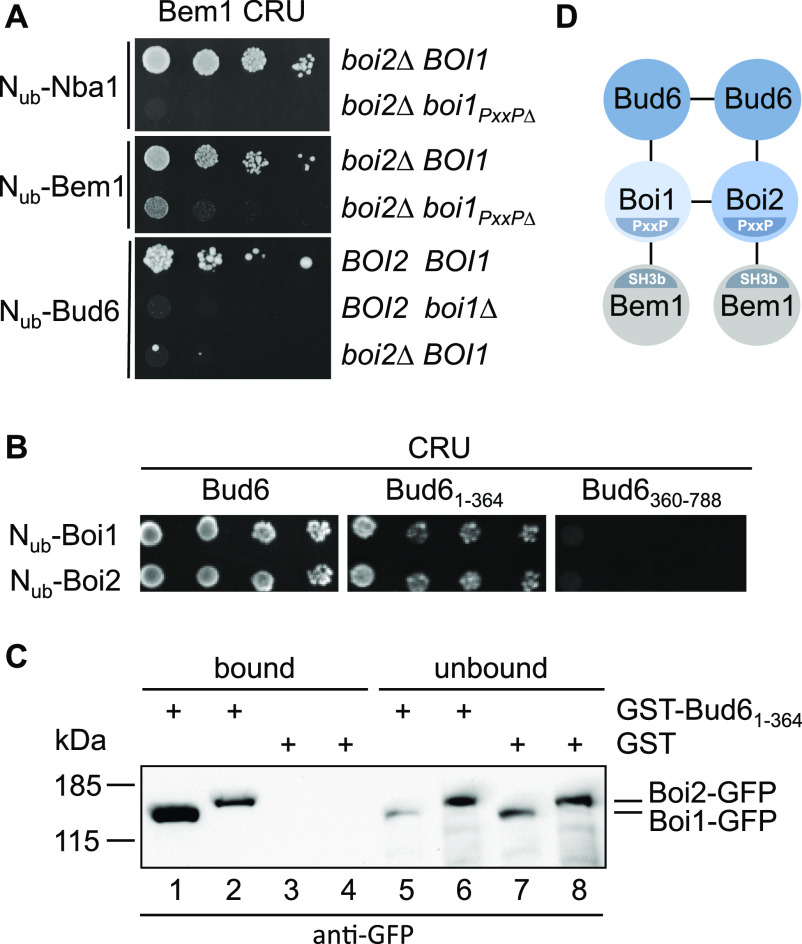
Characterization of the Bem1-Bud6 interaction state. **(A)** Yeast cells carrying the indicated mutations were co-expressing CRU fusions to Bem1 together with the indicated N_ub_ fusions. Cells were grown to an OD_600_ of 1 and spotted in 10-fold serial dilutions onto medium containing 5-fluoro-orotic acid. Interactions are indicated by the growth of the yeast cells. **(B)** As in (A) but with yeast cells co-expressing CRU fusions to Bud6_1-364_ or Bud6_360-788_, together with N_ub_ fusions to Boi1 and Boi2. **(C)** Extracts of yeast cells expressing either Boi1-GFP (lanes 1, 3, 5, and 7) or Boi2-GFP (lanes 2, 4, 6, and 8) were incubated with GST- (lanes 3, 4, 7, and 8) or GST-Bud6_1-364_–immobilized (lanes 1, 2, 5, and 6) sepharose beads. Bound (lanes 1–4) and unbound (lanes 5–8) fractions were analyzed by anti-GFP antibodies after SDS–PAGE and transfer onto nitrocellulose. **(D)** Model of a potential regulator of actin nucleation. Bud6 is known to homodimerize, whereas Boi1 and Boi2 either homo- or heterodimerize. Bud6 binds and stimulates the yeast formin Bni1 (not shown).

The Split-Ub assay detects direct and indirect protein interactions (Hruby et al, 2011; Johnsson, 2014). SH3_b_ mediates the interactions between Bem1 and Boi1, Boi2 (Boi1/2), Nba1, or Bud6 (Fig 1A and B). Boi1/2 bind SH3_b_ and interact directly with Bud6 and Nba1 (Bender et al, 1996; Kustermann et al, 2017). To test whether Boi1/2 link Bem1 to Nba1 or Bud6, we introduced Bem1CRU together with N_ub_-Bud6, or N_ub_-Nba1 in a strain that carried either a deletion of *BOI1* or *BOI2*, or a deletion of *BOI2* and the mutated binding site of Boi1 for Bem1 (*boi2∆ boi1*_*PxxP*∆_). The Split-Ub assays confirmed that the interactions between Bem1 and Bud6 or Bem1 and Nba1 clearly depend on Boi1/2 (Fig 2A). The nearly complete loss of interaction between Bem1CRU and N_ub_-Bem1 in a *boi2∆ boi1*_*PxxP*∆_ strain suggests that the Split-Ub detected Bem1–Bem1 interaction is predominantly mediated by the multimerization of the Boi proteins (Figs 1A and B and 2A) (Kustermann et al, 2017). In contrast to the Nba1-Boi1/2-Bem1 or the Bem1-Boi1/2–Bem1 complex, the interaction between Bud6 and Bem1 was already lost upon deleting either *BOI1* or *BOI2* (Fig 2A).

The Bni1–Bud6 complex nucleates the polymerization of linear actin filaments (Graziano et al, 2011, 2013). Full activity of Bni1 requires its association with a Rho-GTPase (Evangelista et al, 1997; Dong et al, 2003). Bud6 consists of a C-terminal actin- and Bni1-binding domain and an N-terminal region of unknown function (Tu et al, 2012). Testing N_ub_-Boi1 and N_ub_-Boi2 against CRU fusions to the N- and C-terminal fragments of Bud6 located the binding sites for Boi1/2 to its N-terminal 364 residues (Fig 2B). The GST fusion to this fragment precipitated Boi1- and Boi2-GFP from yeast extracts, thus providing an independent confirmation of the Split-Ub analysis and for the existence of a novel potential actin nucleation complex (Fig 2C and D) (Glomb et al, 2020).

**Figure 3.**
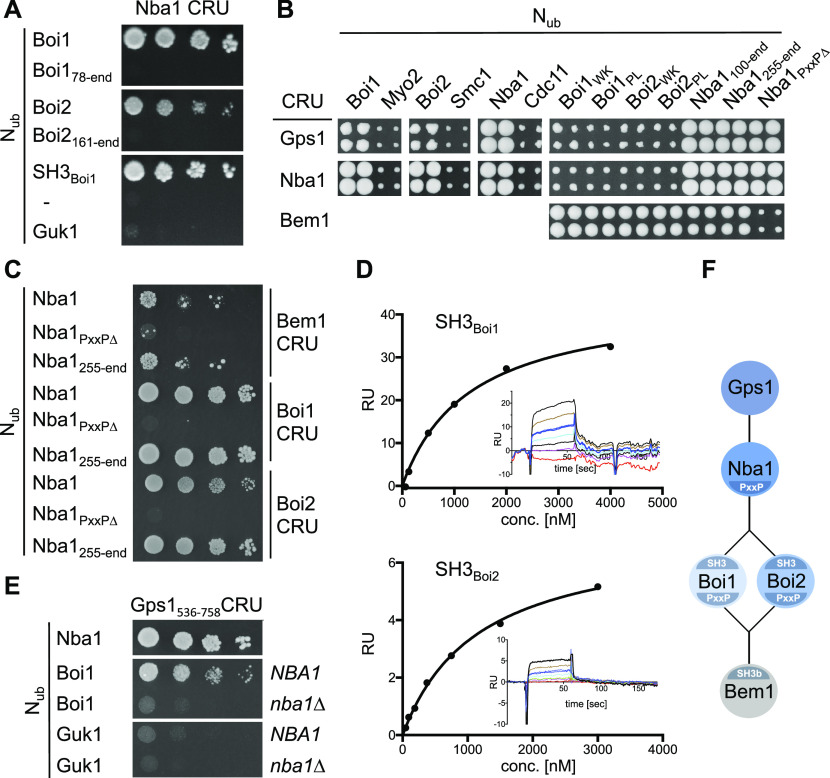
Characterization of the Bem1-Nba1 interaction state. **(A)** Split-Ub assay as in Fig 2A, but with cells co-expressing CRU fusions to Nba1 together with the indicated N_ub_ fusions. N_ub_-Guk1: negative control. **(B)** a-yeast cells expressing the indicated CRU fusions were mated with α-yeast cells expressing the indicated N_ub_ fusions and spotted on 5-fluoro-orotic acid medium as in Fig 1A. **(C)** As in (A) but with yeast cells co-expressing CRU fusions to Bem1, Boi1, or Boi2 together with N_ub_ fusions to Nba1 or its mutants. **(D)** Surface plasmon resonance analysis of the interaction between 6xHIS-Nba1_202-289_-SNAP and the chip-coated SH3 domains of Boi1 or Boi2. Shown are representative plots of the surface plasmon resonance signals as response units against the concentrations of 6xHIS-Nba1_202-289_-SNAP. Corresponding sensograms are shown as insets. **(E)** As in (A) but with yeast cells containing or lacking *NBA1* and co-expressing Gps1_536-758_CRU with the indicated N_ub_ fusions. **(F)** Cartoon of the Nba1-Bem1 interaction state. The postulated indirect interaction between Bem1 and Gps1 was not experimentally observed but inferred from the Nba1-dependent interaction between Boi1/2 and Gps1 (see also Fig 7).

Nba1 was reported to down-regulate the concentration of active Cdc42 during cytokinesis (Meitinger et al, 2014). Testing N- and C-terminal fragments of N_ub_-Boi1/2 located the binding sites for Nba1 to the SH3 domains of both proteins (Fig 3A). Introducing single residue exchanges into the SH3 domains of N_ub_-Boi1/2 (Boi1_WK_, Boi1_PL_, Boi2_WK_, and Boi2_PL_) also abolished the interactions with Nba1CRU (Fig 3B) (Larson & Davidson, 2000). Testing a C-terminal fragment of Nba1 as N_ub_ fusion against Boi1- and Boi2CRU restricted the binding motif for both SH3 domains between residues 256 and 501 of Nba1 (Fig 3B and C). This region harbors a consensus-binding motif for the SH3 domains of Boi1/2 (Tonikian et al, 2009). Removing this PxxP site (Nba1_PxxP∆_, Fig 3C) impaired the interaction between the corresponding N_ub_-Nba1_PxxP∆_ and the C_ub_ fusions of Boi1/2 or Bem1 (Fig 3B and C). Surface plasmon resonance spectrometry determined the K_D_s between the PxxP site (6xHIS-Nba1_202-289_SNAP) and SH3_Boi1_ and SH3_Boi2_ to ∼0.74 μM (n = 3) and 1.97 μM (n = 2), respectively (Fig 3D).

Nba1 is attached to the bud neck through a direct interaction with Gps1 (Meitinger et al, 2014). Split-Ub analysis reproduced the interactions of Gps1CRU with the N_ub_-fusions of Nba1, and Nba1_PxxP∆_, and revealed novel interactions between Gps1CRU and N_ub_-Boi1/2 (Fig 3B). Mutations in the SH3 domains of N_ub_-Boi1/2 abolished their interactions with Gps1CRU (Fig 3B). A C-terminal fragment of Gps1 still interacted as CRU fusion with N_ub_-Nba1 and N_ub_-Boi1 (Fig 3E). The interaction between Gps1_537-758_CRU and N_ub_-Boi1 was lost in an *nba1∆*-strain. The experiments support the existence of a protein complex connecting Bem1 with Gps1 through Boi1/2 and Nba1 (Fig 3E and F).

The interaction signal between Bem1CRU and N_ub_-Cdc11 was lost upon removal of PB1_Bem1_ (Fig 1). Cdc11 interacts directly with Cdc24 (Chollet et al, 2020). We conclude that the interaction between Cdc11 and Bem1 occurs most likely through Cdc24 in a Bem1-Cdc24-Cdc11 complex.

### Functional dissection of Bem1 interaction states

**Figure 4.**
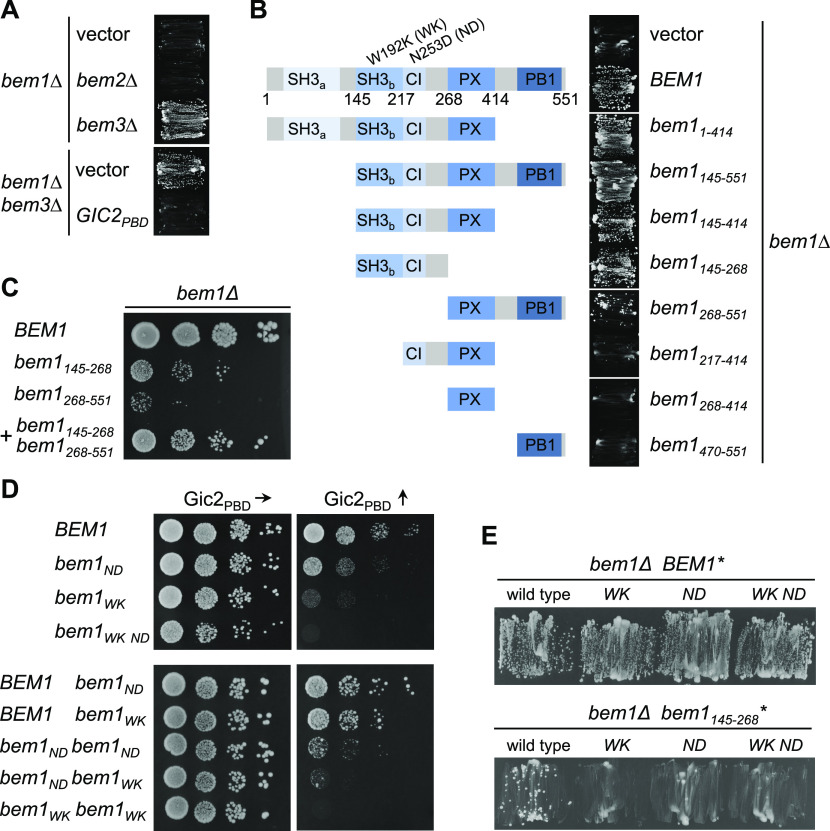
Bem1 contains two functionally independent regions. **(A)** *bem1∆* cells expressing a vector-encoded *BEM1* and carrying an additional gene deletion as well as an empty vector, or a vector expressing Gic2_PBD_, were incubated on media selecting against the presence of the plasmid-encoded *BEM1*. **(B)** *bem1∆* cells carrying a vector-encoded *BEM1* and a vector-expressing Bem1 or the indicated fragments of *BEM1* (left panel) were incubated on medium selecting against the vector-encoded *BEM1* (right panel). **(C)** *bem1∆* cells expressing *BEM1* or the indicated fragments of *BEM1* were spotted in 10-fold serial dilutions onto the SD medium selecting for the presence of the plasmids and incubated at 37°C. **(D)** Haploid cells (upper panel), or diploid cells (lower panel) carrying the indicated alleles of *BEM1* were incubated in 10-fold serial dilutions on media inducing the expression of Gic2_PBD_ to moderate (left panels) or high (right panels) levels. **(E)** *bem1∆* cells carrying a vector-encoded *BEM1* and additionally expressing the full-length *BEM1* with the indicated residue exchanges (upper panel), or fragments of *BEM1* with the indicated residue exchanges (lower panel), were incubated on medium selecting against the vector-encoded *BEM1*.

**Figure S1.**
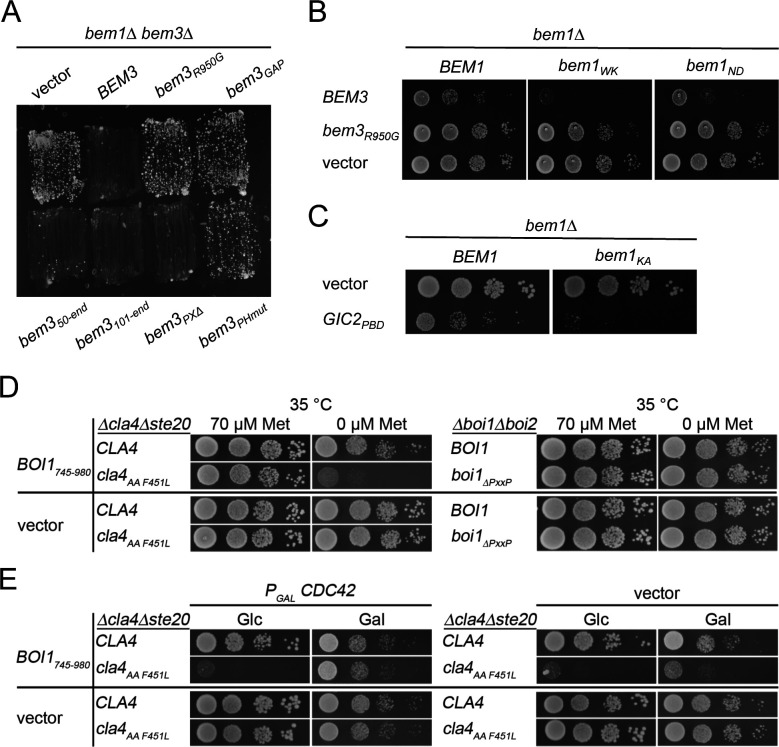
Influence of Cdc42 levels on growth of *bem1*-, *cla4*-, or *boi1*-allele-expresssing cells. **(A)** *bem1∆bem3∆* cells harboring *BEM1* with its native promotor on a centromeric, *URA3*-expressing plasmid and containing the indicated GFP fusion alleles of *BEM3* on an additional plasmid were streaked on medium containing 5-fluoro-orotic acid and selecting for the presence of the *bem3* containing plasmids. Growth of the cells indicates alleles that do not complement the function of *BEM3*. *bem3*_*PHmut*_ harbors the residue exchanges R646S, R645S, and K647D that are known to impair the binding of the PH domain to phospholipids (Mukherjee et al, 2013). *bem3*_*R950G*_ contains an exchange in the catalytic site of the GAP domain that eliminates the activity of Bem3. **(B)** Cells containing the indicated *bem1* alleles and an empty vector or overexpressing Bem3-GFP or Bem3_R950G_-GFP from a centromeric plasmid under the control of the P_*MET17*_ promoter were spotted in 10-fold serial dilutions on media without methionine to induce the expression of the *bem3* alleles. Cells were incubated for 2 d at 37°C. **(C)** *bem1∆* cells expressing *BEM1* or *bem1*_*KA*_ from a centromeric plasmid under control of the P_*MET17*_ promoter and containing an empty vector (upper lanes), or Gic2_PBD_ expressed from the P_*MET17*_-promoter (lower lanes), were spotted in 10-fold serial dilutions on medium-lacking methionine and incubated for 2 d at 30°C. *bem1*_*KA*_ harbors the residue exchanges K482A that is known to impair the binding of Cdc24. **(D)** Left panel: *cla4∆ ste20∆* cells expressing *CLA4* or *cla4*_*AAF451L*_ and either the PH domain of Boi1 (Boi1_745-980_) or an empty vector were spotted on plates containing 70 μM methionine or no methionine to fully express Boi1_745-980_. Right panel: *boi1∆ boi2∆* cells expressing *Boi1* or *boi1*_*∆PxxP*_ and either the PH domain of Boi1 (Boi1_745-980_) or an empty vector were spotted on plates containing 70 μM methionine or no methionine to fully express Boi1_745-980_. Cells were incubated for 2 d at 35°C. **(D, E)** Cells as in left panel of (D) but additionally expressing Cdc42 on a centromeric vector under the control of the P_*GAL1*_ promoter (left panel) or containing an empty vector (right panel). Cells were spotted on media containing glucose (Glc) and lacking methionine to overexpress Boi1_745-980_ and repress Cdc42 or on media lacking methionine and containing galactose (Gal) to simultaneously overexpress Boi1_745-980_ and Cdc42. Note the partial rescue of *cla4*_*AAF451L*_ cells overexpressing Cdc42.

Bem1 is thought to coordinate the activities of its ligands by bringing them into close spatial proximity. To define which combination of binding sites and partners constitute the essential configurations of the Bem1 complex, we first tested mutants and fragments for their ability to complement a deletion of *BEM1*. *BEM1* is not essential in all yeast strains but is required for cell survival in the strain JD47 (Fig 4A) (Dowell et al, 2010). *bem1∆* cells can be rescued by a deletion of the Cdc42 GAP Bem3 but not by the deletion of the Cdc42 GAP Bem2 (Fig 4A) (Laan et al, 2015). Mutating Bem3’s GAP domain or impairing its interaction with lipids was sufficient to restore viability to *bem1∆* cells (Fig S1A). We conclude that a high concentration of cortical Cdc42_GTP_ can compensate for the loss of Bem1.

The CRIB domain of Gic2 (Gic2_PBD_) interacts with Cdc42_GTP_ and has been tagged with red fluorescent protein to monitor active Cdc42 in living yeast cells (Brown et al, 1997; Orlando et al, 2008; Atkins et al, 2013; Okada et al, 2013). The overexpression of Gic2_PBD_ is toxic in certain yeast mutants and can be compensated by increasing the amount of Cdc42 (Brown et al, 1997). We introduced Gic2_PBD_ under control of the methionine-sensitive P_*MET17*_*-* promoter in *bem1∆ bem3∆* cells. Omitting methionine in the media increased the expression of Gic2_PBD_ and eliminated the positive effect of the *BEM3* deletion on the survival of *bem1∆* cells (Figs 4A and S4A). The results imply that Gic2_PBD_ might reduce the free pool of Cdc42_GTP_ at the cell cortex. It follows that Bem1 is needed to stimulate the synthesis and/or to improve the effective use of this pool.

**Figure S2.**
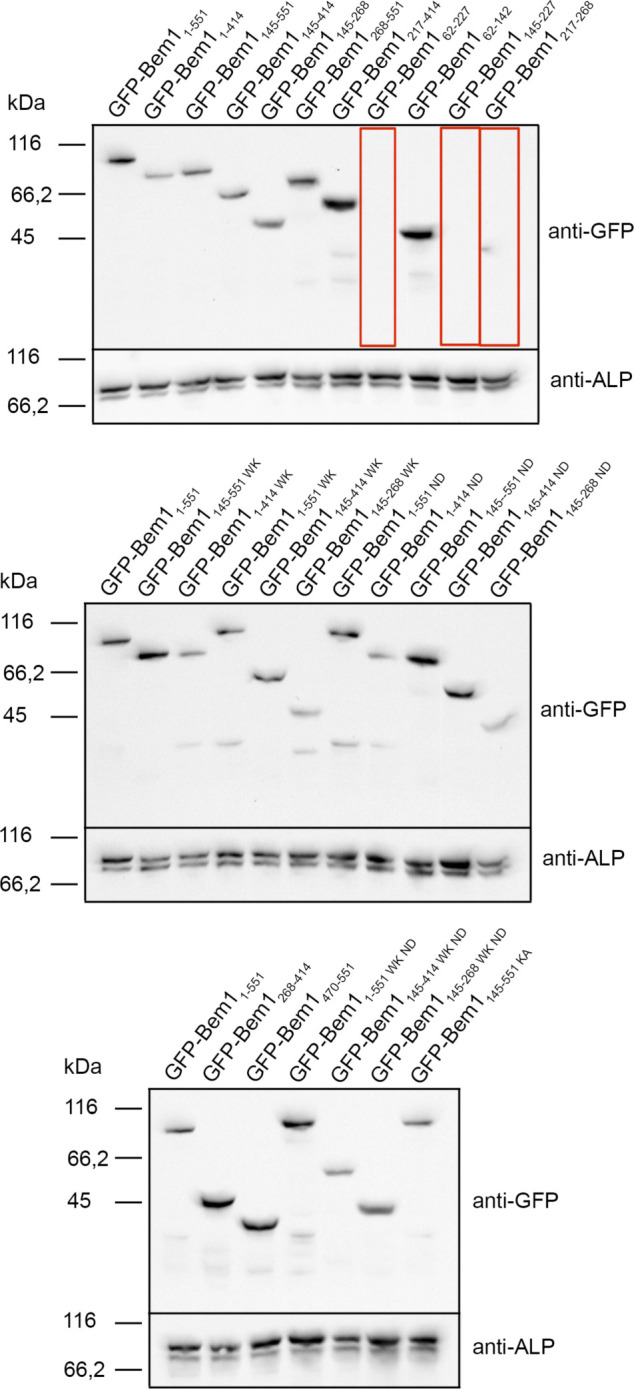
Expression levels of different *BEM1* alleles. N-terminal GFP-tagged fragments of Bem1 and mutants thereof were expressed under control of the P_*MET17*_ promoter from a centromeric plasmid. Yeast cells were grown in media containing 70 μM methionine to an OD_600_ of 1.5–2. Extracts containing equal amounts of proteins were separated by SDS–PAGE and stained with anti-GFP antibody after transfer onto nitrocellulose. Equal loading was controlled by staining the blot with anti-alkaline phosphatase antibody (anti-ALP). Lanes showing fragments that are not measurably expressed were framed by a red box.

A fragment of Bem1 (Bem1_145-551_) that covers SH3_b_CI, the PX-, and the PB1 domain and thus keeps the majority of all detected interactions, complemented *bem1∆* cells (Figs 4B and S2). This region could be further divided into two independently complementing fragments: the SH3_b_CI domain (Bem1_145-268_) with its binding sites for Cdc42 and for its PxxP ligands Ste20, Cla4, Boi1/2, and the C-terminal fragment containing the PX- and the PB1 domain (Bem1_268-551_) with its binding sites for Cdc24, lipids, and the other PB1-domain ligands (Figs 1A, 4B and C, and S2). Expressing both fragments together complemented *bem1∆* cells much better than each fragment alone (Fig 4C).

The autonomy of the central SH3_b_CI-domain was unexpected. Single mutations that interrupt the binding of SH3_b_CI either to the PxxP ligands (SH3_bWK_CI) or to Cdc42_GTP_ (SH3_b_CI_ND_) interfered with the fragment’s ability to rescue *bem1∆* cells (Figs 4E and S2) (Yamaguchi et al, 2007; Gorelik & Davidson, 2012).

The existence of two independently complementing regions explains why none of the single mutations in the SH3_b_CI domain or the deletion of the PB1 domain eliminated the functionality of the otherwise full-length Bem1 (Fig 4B and E). By expressing increasing amounts of Gic2_PBD_, we tested the functionality of the *bem1*_*WK*_-, *bem1*_*KA*_-, *bem1*_*ND*_-, or *bem1*_*WK ND*_ alleles under conditions of limiting Cdc42 (Fig 4D). All interaction-interfering mutations drastically decreased the tolerance of the cells toward Gic2_PBD_ overexpression. The allele *bem1*_*WK ND*_ bearing both mutations in the SH3_b_CI domain conferred a higher sensitivity than the singly mutated *bem1*_*WK*_- or *bem1*_*ND*_ allele. Overexpressing Bem3 and thus reducing Cdc42_GTP_ at the cortex by different means had a similar impact (Fig S1B).

We introduced the WK and ND mutations in the different *BEM1* copies of a diploid cell to test trans-complementation of the co-expressed Bem1_WK_ and Bem1_ND_. The undiminished sensitivity of these cells toward Gic2_PBD_ overexpression showed that complementation does not occur in trans and that both binding sites operate within the same Bem1 molecule (Fig 4D).

### Functional annotation of the SH3_b_ interactions

**Figure S3.**
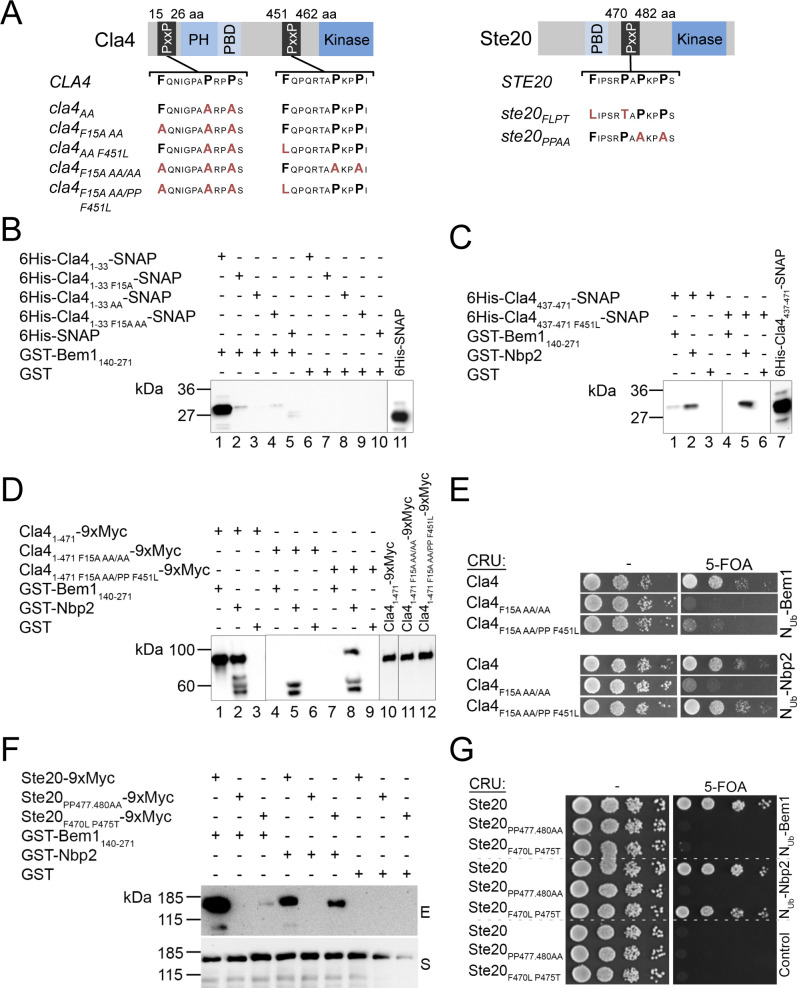
Characterization of mutations in Cla4 and Ste20 that impair the interactions with Bem1 but not with Nbp2. **(A)** Cartoons of Cla4 (left panel) and Ste20 (right panel) indicating the positions of the kinase domains and the motifs for binding Cdc42_GTP_ (PBD) and Bem1 (PxxP). Below are indicated the studied alleles of the PAKs and their corresponding residue exchanges. **(B)** Extracts of *E. coli* cells expressing a 6His–SNAP fusion protein of the N-terminal PxxP-motif of Cla4 (6His-Cla4_1-33_-SNAP) containing no mutation (lanes 1, 6), a phenylalanine to leucine exchange at position 15 (6His-Cla4_1-33F15A_-SNAP; lanes 2, 7), an alanine to proline exchange at positions 22 and 25 (6His-Cla4_1-33AA_-SNAP; lanes 3, 8), the triple mutations (6His-Cla4_1-33F15AAA_-SNAP; lanes 4, 9), or a 6His-SNAP fusion (lanes 5, 10) were incubated with Glutathione-coated beads exposing GST-Bem1_140-271_ (lanes 1–5), or GST (lanes 6–19). Shown is the anti-His Western blot of the glutathione eluates of the beads after SDS–PAGE and transfer onto nitrocellulose. Lane 11 documents the input of the 6His–SNAP fusion. **(C)** Analysis as in (B) but with extracts of *E. coli* cells expressing a 6His–SNAP fusion protein of C-terminal PxxP-motif of Cla4 (6His-Cla4_437-471_-SNAP) containing no mutation (lanes 1–3) or a phenylalanine to leucine exchange at position 451 (6His-Cla4_437-471F451L_-SNAP; lanes 4–6). Extracts were incubated with beads exposing GST-Bem1_140-271_ (lanes 1, 4), GST-Nbp2 (lanes 2, 5), or GST (lanes 3, 6). The input is shown in lane 7. **(D)** A Myc-tagged fragment of Cla4 containing both binding sites and no mutations (Cla4_1-471_9xMyc; lanes 1–3), the indicated mutations (Cla4_1-471F15A AA/AA_; lanes 4–6), or Cla4_1-471F15A AA/PP F451L_ (lanes 7–9) were expressed in yeast from a centromeric vector under control of the P_*MET17*_ promoter. Extracts of these cells were incubated with bead-coupled GST-Bem1_140-271_ (lanes 1, 4, and 7), GST-Nbp2 (lanes 2, 5, and 8), or GST (lanes 3, 6, and 9). The inputs of the Myc-tagged fusions are shown in lanes 10–12. The eluted proteins were separated by SDS–PAGE and stained with anti-Myc antibody after transfer onto nitrocellulose. The protein doublet below 60 kD is occasionally observed in lanes displaying GST-Nbp2 and might arise by a cross-reactivity of the antibodies. **(E)** Split-Ub assay of yeast cells co-expressing Nu_b_-Bem1 (upper panel) or Nu_b_-Nbp2 (lower panel) together with CRU fusions to Cla4 or its indicated mutants, expressed from a centromeric vector under control of the P_*MET17*_ promoter. 4 μl of cells of OD_600_ of 1 was spotted in 10-fold serial dilutions on media lacking (left panels) or containing 5-fluoro-orotic acid. Growth on media containing 5-fluoro-orotic acid indicates interaction between the respective Nu_b_ and CRU fusion. **(F)** As in (D) but with cells co-expressing 9xMyc fusions to Ste20, to Ste20 containing alanine exchanges for proline at positions 477 and 489 (Ste20_PP477,480AA_) or to Ste20 containing an phenylalanine to leucine exchange at position 470 and a proline to threonine exchange at position 475 (Ste20_F470L P475T_). E, Glutathione eluate; S, Supernatant. **(E, G)** Split-Ub assay as in (E) but with cells co-expressing the indicated Nu_b_ fusions with CRU fusions to Ste20 or to the indicated mutants of Ste20. The CRU fusions were expressed from a centromeric vector under the control of the P_*MET17*_ promoter.

**Figure 5.**
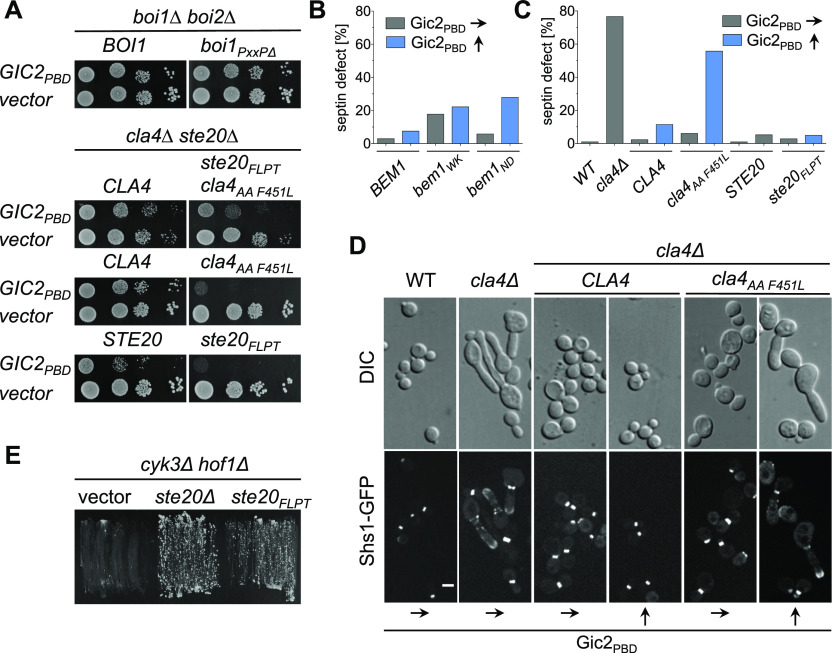
The connection between Bem1 and the PAKs is essential upon overexpression of Gic2_PBD_. **(A)** Yeast cells carrying the indicated alleles and either Gic2_PBD_ or an empty vector were spotted in 10-fold serial dilutions on medium inducing high levels of Gic2_PBD_. **(B)** Cells expressing Shs1-GFP, the indicated alleles of *BEM1*, and Gic2_PBD_ were incubated under conditions of low (70 μM Met, gray bars) or high expression levels (0 Met, blue bars) of Gic2_PBD_. Cells (500 < n < 600) were classified according to their native-like or abnormal distribution of the Shs1-GFP. **(C)** As in (B) but with cells (500 < n < 600) carrying the indicated alleles of *STE20* or *CLA4*. **(D)** Microscopy of the cells of (C). Upper panel: DIC channel. Lower panel: GFP channel. Scale bar indicates 3 μM. **(E)** *cyk3∆ hof1∆* cells expressing *HOF1* from an extra-chromosomal vector and carrying the indicated alleles of *STE20* or an empty vector were incubated on media selecting against the *HOF1*-containing vector.

**Figure S4.**
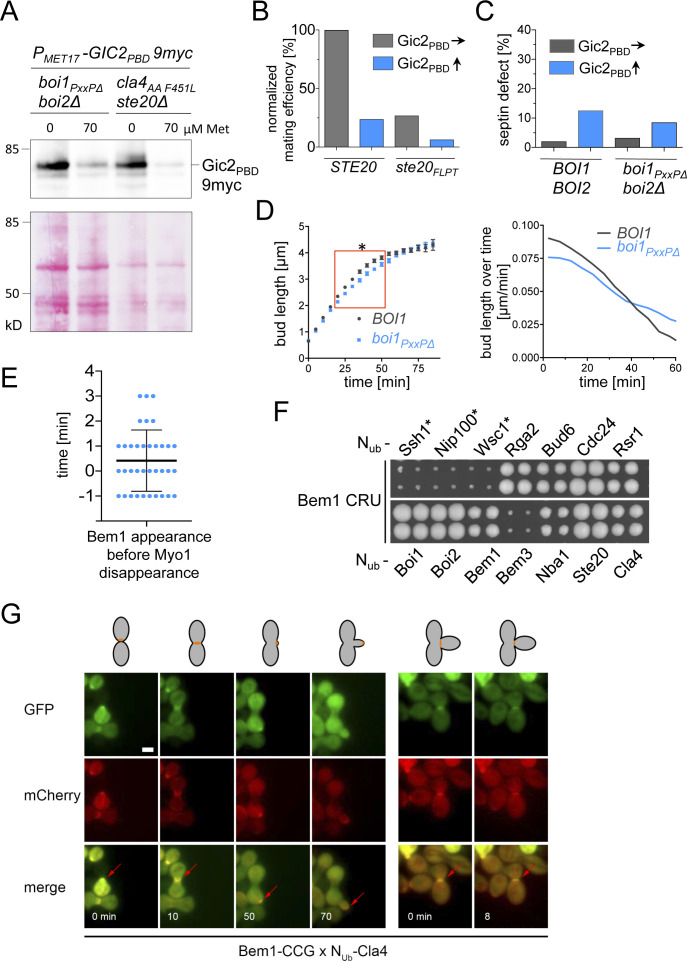
Phenotypes of *boi1*, or *ste20*-allele-expressing cells. **(A)** Cells of the indicated genotypes and expressing Gic2_PBD_-9myc under control of the P_*MET17*_ promoter were grown to an OD_600_ of 1 in medium containing 70 μM Met. Cells were spun down and resuspended in fresh medium containing either no or 70 μM Met. After 4 h of growth, equal cell numbers were lysed by boiling in SDS sample buffer, and the extracts were separated by SDS–PAGE. Shown are the anti-Myc antibody staining (upper panel) and Ponceau staining (lower panel) of the gels after transfer onto nitrocellulose. **(B)** a- and α-cells containing the same alleles of *STE20* and expressing Gic2_PBD_ were incubated together in media inducing low (70 μM Met, blue bars) or high expression (0 Met, gray bars) of Gic2_PBD_ and plated on media selecting for the presence of diploid cells. **(C)** The disruption of the Boi1/2-Bem1 connection does not induce defects in septin organization. Quantification of wild type and *boi2∆ boi1*_*PxxP*∆_ cells as in Fig 5C. 500–600 cells containing a GFP fusion to the septin *SHS1* were inspected under the fluorescence microscope under conditions of low (70 μM methionine, blue bar) or high (no methionine, gray bar) expression of Gic2_PBD_. No increased septin mislocalization is detectable in *boi2∆ boi1*_*PxxP*∆_ cells. **(D)** The bud length of cells (n = 38; SEM) carrying the indicated alleles of *BOI1* and co-expressing Bem1-GFP and Shs1-mCHERRY were measured every min starting with a bud length of 0.65 μm and ending at the time point of septin splitting (left panel). Red box indicates the time window with a significant difference between *∆boi2 BOI1*- and *∆boi2 boi1*_*PxxP*∆_ cells. Right panel shows the first derivatives of the growth curves of the left panel and compares the rates of bud extension in μm/min. **(E)** Time-lapse analysis of cells co-expressing Bem1-GFP and Myo1-mCherry. Shown are the quantifications of 36 cells. Plotted are the times of Bem1-GFP appearances in relation to the disappearance of Myo1-mCherry. Bem1 appears in average 0.4 min before Myo1 is removed from the site of cell separation. **(F)** Split-Ub analysis as in Fig 1 but of cells expressing Bem1CRU together with the indicated N_Ub_ fusions expressed from their native promoters. * indicates Nu_b_ fusions expressed from the P_*CUP1*_ promoter. Interaction was scored after 4 d of growth on media containing 5-fluoro-orotic acid. **(G)** SPLIFF analysis of a diploid cell formed by the fusion of a Bem1CCG-expressing a-cell and a Nu_b_-Cla4–expressing α-cell. Shown are the stills from the different cell cycle phases of cell fusion, bud formation, bud growth, and cell separation (see cartoon at the top). Red arrows point to regions where the fluorescence intensities were quantified. Note that the α-cell is not visible before cell fusion has occurred (first frame, 0 min).

Our findings imply that delivering Cdc42_GTP_ to one or more of its SH3_b_ ligands constitutes the essential activity of Bem1. Which is the essential binding partner of SH3_b_? The closely spaced SH3_b_ and CI domains of Bem1 mirror the Cdc42_GTP_ binding of the three of its four ligands Cla4, Ste20, and Boi1. The binding of Cdc42 to Boi2 was not yet investigated. None of the four SH3_b_ ligands are essential. Cells, however, do not tolerate the loss of both PAKs, or of both Boi-proteins (Cvrckova et al, 1995; Bender et al, 1996). To identify the interactions whose loss could phenocopy the WK mutation in SH3_b_, we introduced mutations in *CLA4* (*cla4*_*F15AAA/PPF451L*_ = *cla4*_*PPAFL*_), *STE20* (*ste20*_*F470L P475T*_ *= ste20*_*FLPT*_), and *BOI1* (*boi1*_*PxxP*∆_) that specifically reduce their affinities to SH3_b_ (Bender et al, 1996; Kozubowski et al, 2008; Gorelik & Davidson, 2012) (Fig S3). The mutations in *STE20* and *CLA4* did not impair their interaction with Nbp2, a further ligand of their PxxP motifs (Fig S3) (Winters & Pryciak, 2005; Hruby et al, 2011; Gorelik & Davidson, 2012). Which of the SH3_b_ interactions become essential under conditions of Gic2_PBD_ overexpression? Gic2_PBD_ overexpression killed cells lacking *CLA4* and the SH3_b_-binding motif of Ste20, or cells lacking *STE20* and the SH3_b_-binding motifs of Cla4, or cells co-expressing *cla4*_*PPAFL*_ with *ste20*_*FLPT*_ (Figs 5A and S4A). Cells lacking *BOI2* and the Bem1-binding site in Boi1 were not affected by Gic2_PBD_ overexpression (Fig 5A). The pleckstrin homology (PH) domain of Boi1 (PH_Boi1_) binds lipid and Cdc42_GTP_ (Bender et al, 1996; Kustermann et al, 2017). Overexpression of PH_Boi1_ killed cells lacking the physical connection of Bem1 to Ste20 and Cla4 but does not affect cells lacking the connection of Bem1 to Boi1/2 (Fig S1D). A simultaneous overexpression of Cdc42 suppressed the toxic effect of PH_Boi1_ on *ste20∆ cla4*_*PPAAFL*_ cells (Fig S1E). We conclude that the PAKs are the essential ligands of the SH3_b_ domain of Bem1 under conditions of limiting concentrations of active Cdc42.

Cells without Cla4 or its kinase activity do not correctly assemble septins and display elongated buds (Holly & Blumer, 1999; Weiss et al, 2000). These phenotypes were recapitulated in *bem1*_*WK*_- or in *bem1*_*ND*_ cells, or in *cla4*_*PPAAFL*_ cells upon overexpression of Gic2_PBD_ (Fig 5B–D). In contrast, Gic2_PBD_ overexpression did not affect cellular morphology or septin structure of *ste20*_*FLPT*_- or *boi2∆ boi1*_*PxxP*∆_ cells (Figs 5C and S4C). The experiments prove that the Cla4–Bem1–Cdc42_GTP_ complex is important during incipient bud site- and septin-assembly.

A deletion of *STE20* rescues a strain that is arrested at cytokinesis by the loss of the cytokinesis factors Hof1 and Cyk3 (Atkins et al, 2013; Onishi et al, 2013). It is speculated that Cdc42 inhibits secondary septum formation through activation of Ste20. The loss of Ste20 might prematurely activate secondary septum formation, thus compensating the lack of primary septum. Fig 5E shows that *ste20*_*FLPT*_ also rescues *hof1∆ cyk3∆* cells. Accordingly, the Ste20–Bem1 complex is functionally relevant during cell separation. The same interaction is important for the fusion of cells during mating (Fig S4B) (Winters & Pryciak, 2005). Again, overexpression of Gic2_PBD_ potentiated the effect of the *ste20*_*FLPT*_ allele (Fig S4B).

The Boi proteins stimulate the fusion of secretory vesicles with the plasma membrane (Kustermann et al, 2017; Masgrau et al, 2017). The small difference in the rate of bud length extension between *boi2∆* and *boi2∆ boi1*_*PxxP*∆_ cells suggests that the interactions between Boi1/2 and Bem1 only modestly affect this activity (Fig S4D).

### Boi1/2 anchor Bem1 at the bud tip

**Figure 6.**
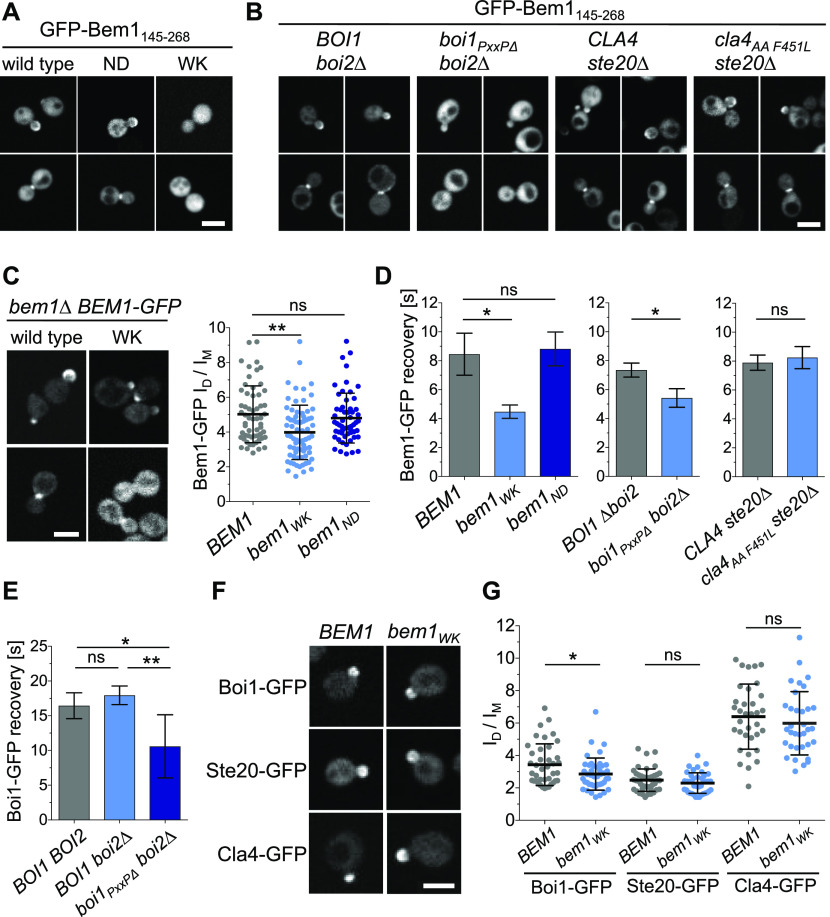
Boi1 and Boi2 localize Bem1-Cdc24 at bud tip and neck. **(A)** Wild-type cells expressing GFP-Bem1_145-268_ (left panel) or GFP-Bem1_145-268_ carrying the ND (middle panel) or WK (right panel) exchange were inspected by fluorescence microscopy. **(B)** Cells of the indicated genotypes expressing GFP-Bem1_145-268_ were inspected by fluorescence microscopy (left panel). Only *boi2∆ boi1*_*PxxP*∆_ cells show a clear misdistribution of GFP-Bem1_145-268_. **(C)** Left panel: *bem1∆* cells expressing Bem1-GFP or Bem1_WK_-GFP were inspected by fluorescence microscopy. Right panel: Quantification of the intensity ratios of Bem1-GFP (n = 58), Bem1_WK_-GFP (n = 72), and Bem1_ND_-GFP (n = 57) in bud and mother cells. **(D)** Half-times of fluorescence recovery after photo-bleaching the bud of cells expressing Bem1-GFP or its mutants. Left panel: Bem1-GFP (n = 11), Bem1_WK_-GFP (n = 18), or Bem1_ND_-GFP (n = 24). Middle panel: Bem1-GFP in *boi2∆ BOI1* cells (n = 20) or *boi2∆ boi1*_*∆PxxP*_ cells (n = 16). Right panel: Bem1-GFP in *ste20∆ CLA4* cells (n = 16) or *ste20∆ cla4*_*PPAAFL*_ cells (n = 14). **(E)** As in (D) but with *BOI1 BOI2 cells* (n = 23), *boi2∆ BOI1* cells (n = 24), or *boi2∆ boi1*_*PxxP*∆_ cells (n = 22) expressing GFP fusions to *BOI1* or *boi1*_*PxxP*∆_. **(F)** Bem1 cells (left panel), or Bem1_WK_ cells (right panel) expressing GFP fusions to Boi1 (upper row), Ste20 (middle row), or Cla4 (lower row), were inspected by fluorescence microscopy. **(F, G)** The ratios of the fluorescence intensities of bud and mother cells from (F) were quantified in *BEM1*- and *bem1*_*WK*_ cells expressing Boi1-GFP (n = 40, 43), Ste20-GFP (n = 43, 40), or Cla4-GFP (n = 34, 34). ns, not significant. * = *P* < 0.05, ** = *P* < 0.01. Scale bars indicate 3 μM.

SH3_b_CI-GFP is the minimal fragment that fully recapitulates the cellular distribution of Bem1-GFP (Fig 6A). The cortical targeting of SH3_b_CI required the ligands of SH3_b_ as SH3_bWK_CI-GFP stayed cytosolic throughout the cell cycle (Fig 6A). SH3_b_CI_ND_-GFP was still concentrated at bud neck and tip. Under the assumption that the N253D exchange completely abrogates Cdc42_GTP_ binding, we conclude that Cdc42 does not contribute to the cortical localization of SH3_b_CI. To find out which of the four SH3_b_ ligands influences the distribution of SH3_b_CI-GFP, we expressed SH3_b_CI-GFP in cells each lacking a specific SH3_b_-binding site. The analysis identified Boi1/2 as the receptor for SH3_b_CI at the cortex and bud neck (Fig 6B).

**Figure 7.**
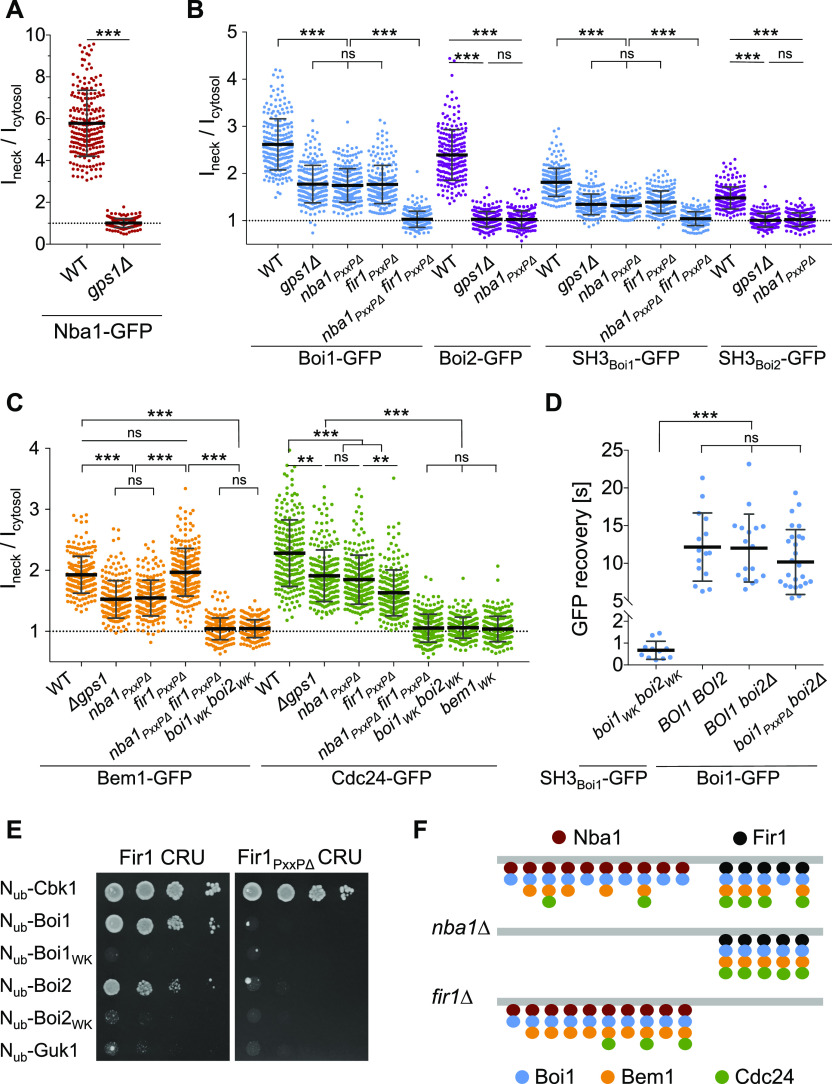
Two receptor systems attach Bem1-Cdc24 to the bud neck. **(A)** Ratios of bud neck to cytosolic fluorescence intensities of Nba1-GFP in wild type and *gps1∆* cells. **(B)** As in (A) but in cells of the indicated genotypes expressing Boi1-GFP, Boi2-GFP, SH3_Boi1_-GFP, and SH3_Boi2_-GFP. **(C)** As in (A) but in cells of the indicated genotypes expressing Bem1-GFP or Cdc24-GFP. **(D)** Fluorescence recovery after photo bleaching the bud neck of *boi1*_*WK*_ *boi2*_*WK*_ cells expressing SH3_Boi1_-GFP (n = 11), of wild type cells expressing Boi1-GFP (n = 15), of *boi2∆* cells expressing Boi1-GFP (n = 17), of *boi2∆* cells expressing Boi1_*PxxP*∆_-GFP (n = 27). **(E)** Split-Ub analysis as in Fig 2A but with cells expressing Fir1CRU or Fir1_PxxP∆_CRU together with indicated N_ub_ fusions. N_ub_-Cbk1: positive control; N_ub_-Guk1: negative control. **(F)** Anchoring the Bem1–Cdc24 complex at the bud neck in wild type- (upper panel), *nba1∆-* (middle panel), and *fir1∆* cells (lower panel). Nba1 (red dots) and Fir1 (black dots) recruit Boi1 (blue dots) (for simplicity only Boi1 is shown), which recruits the Bem1–Cdc24 complex (orange and green dots). To explain the impact of the mutations on the distributions of the different proteins, we assume that Nba1 outnumbers Fir1 at the neck, that Nba1 and Fir1 are saturated by Boi1/2, the number of Nba1 and Bem1 molecules are similar, and that Nba1 reduces the affinity between Cdc24 and Bem1 (Meitinger et al, 2014).

Bem1_WK_-GFP was barely detected at the bud neck (Fig 6C, see also Fig 7), whereas its fluorescence signal at the tip of small and large buds was only modestly reduced (Fig 6C). To obtain a more quantitative measure of tip adherence, we compared the FRAPs between the cortex-localized Bem1-GFP, Bem1_WK_-GFP, and Bem1_ND_-GFP (Fig 6D). The halftime of recovery was not changed by the N253D mutation in Bem1 (t_1/2_ = 8.82 ± 1.12 s), whereas the W192K exchange reduced t_1/2_ to 4.48 ± 0.46 s (Fig 6D). A similar reduction in t_1/2_ was observed when the FRAPs of Bem1-GFP were compared between *boi2∆* cells (t_1/2_ = 7.35 ± 0.49 s) and *boi2∆* cells lacking the Bem1-binding sites in Boi1 (t_1/2_ = 5.42 ± 0.49 s) (Fig 6D). The FRAP of the cortex-localized Bem1 was not changed in cells where the interactions between Bem1 and the PAKs were eliminated (*ste20∆ cla4*_*PPAAFL*_) (Fig 6D).

Boi1 and Boi2 associate with the bud cortex mainly through their Cdc42_GTP_- and lipid-binding PH domains (Hallett et al, 2002). Bem1 also contains multiple phospholipid-binding sites (Hallett et al, 2002; Meca et al, 2019). Boi1/2 and Bem1 might, thus, cooperatively recruit each other to the bud cortex. In accordance, Boi1_PxxP∆_-GFP carrying a mutated Bem1-binding site was significantly more mobile than the native protein (t_1/2_ = 16.43 ± 1.86 s versus t_1/2_ = 10.58 ± 0.97 s) (Fig 6E). Boi1-GFP was also slightly less focused at the bud cortex of *bem1*_*WK*_ cells. *bem1*_*WK*_ did not detectably influence the cortical localization of Ste20-GFP or Cla4-GFP (Fig 6F and G) (Winters & Pryciak, 2005).

### Nba1 and Fir1 anchor Boi1/2-Bem1-Cdc24 at the bud neck

Bem1 leaves the cortex during mitosis and arrives at the bud neck shortly before the acto-myosin ring contraction is completed (Fig S4E). Boi1/2 link Bem1 and Cdc24 to the neck (Figs 6B and 7C), as mutations in their SH3 domains removed both proteins from the neck (Fig 7C) (Hallett et al, 2002). Nba1 is a potential docking site for Boi1/2 as it binds to both SH3 domains and arrives at the neck at roughly the same time as Bem1 (Fig 3) (Meitinger et al, 2014). Accordingly, a deletion of *NBA1* or of its Boi1/2-binding site (Nba1_PxxP∆_) removed Boi2-GFP completely, and 55% of Boi1-GFP (Fig 7B). The Nba1-mediated interaction between Gps1 and Boi1/2 suggests that Gps1 anchors the Nba1–Boi1/2–Bem1–Cdc24 complex at the neck (Fig 3F and E). Accordingly, *gps1∆* cells lacked Nba1-GFP at the bud neck and reduced neck localizations of Boi1-GFP and Boi2-GFP to a similar extent as *nba1∆* cells (Fig 7A and B) (Meitinger et al, 2014). The localization of the isolated SH3_Boi1_ or SH3_Boi2_ mirrored the SH3 dependencies of the full-length proteins (Fig 7B). However, t_1/2_ of FRAP of SH3_Boi1_-GFP was significantly shorter than t_1/2_ of the full-length Boi1-GFP (t_1/2_ = 0.7 s versus t_1/2_ = 12 s; Fig 7D), indicating that regions beyond SH3_Boi1_ contribute to neck localization. Bem1 was reported to directly bind to Nba1 (Meitinger et al, 2014). However, Boi1_PxxP∆_-GFP lacking the binding site to Bem1 still displayed a t_1/2_ of 10 s at the bud neck that is very similar to the t_1/2_ of FRAP of the wild-type protein (Fig 7D). We conclude that Boi1 recruitment to the neck is distinct from its synergistic recruitment to the tip.

Bem1-GFP and Cdc24-GFP completely disappeared from the bud neck of cells expressing SH3 mutations in both Boi proteins (*boi1*_*WK*_ *boi2*_*WK*_, Fig 7C). In contrast, *nba1*_*PxxP*∆_- or *gps1∆* cells still kept ∼69% of Cdc24-GFP and 58% of Bem1-GFP at the neck (Fig 7C). Whereas Boi2 was solely attached by Nba1-Gps1, 45% of Boi1 were still visible in *nba1*_*PxxP*∆_-, or *gps1∆* cells.

Which protein is the alternative anchor for Boi1 at the site of cell separation? The neck localization as well as its interaction with Boi1/2 makes Fir1 a candidate for this role (Brace et al, 2019; Tonikian et al, 2009). Split-Ub analysis confirmed the complex between Boi1/2 and Fir1 and could further demonstrate that the interactions depend on the functional SH3_Boi1_ or SH3_Boi2_ and the predicted SH3_Boi1/2_-binding motif in Fir1 (Fig 7E) (Tonikian et al, 2009). We next introduced the GFP fusions of Boi1, SH3_Boi1_, and the Cdc24–Bem1 complex in strains lacking *FIR1* (*fir1∆*), lacking the Boi1-binding motif in *FIR1* (*fir1*_*PxxP*∆_), or in cells lacking the motifs in *FIR1* and *NBA1* (*nba1*_*PxxP∆*_ *fir1*_*Pxx*P∆_) (Fig 7B and C). Quantifying the intensities of the GFP signals proved that Fir1 recruits the Boi1–Bem1–Cdc24 complex independently of Nba1 to the bud neck. Boi1 was equally distributed between Nba1 and Fir1 (Fig 7B). In contrast, proportionally more of Cdc24 was anchored through Fir1 than through Nba1, whereas the amount of neck-anchored Bem1 did not change upon removal of the Boi1-binding site in Fir1 (Fig 7C and F for a model).

### Temporal dissection of the Bem1 interaction network

To find out whether Bem1 might bring together different interaction partners at different phases of the cell cycle, we characterized the time dependency of a subset of its interactions through Split-Ub analysis using two spectrally different fluorescent proteins as sensors for interaction (SPLIFF) (Moreno et al, 2013). Here, the C_ub_ is sandwiched between the auto-fluorescent mCherry and GFP (CCG) (Moreno et al, 2013). Upon interaction-induced reassociation with an N_ub_ fusion, the GFP is cleaved off and rapidly degraded. The subsequent local increase in the ratio of red to green fluorescence indicates where and when the direct or indirect interaction between both proteins took place (Moreno et al, 2013).

**Figure 8.**
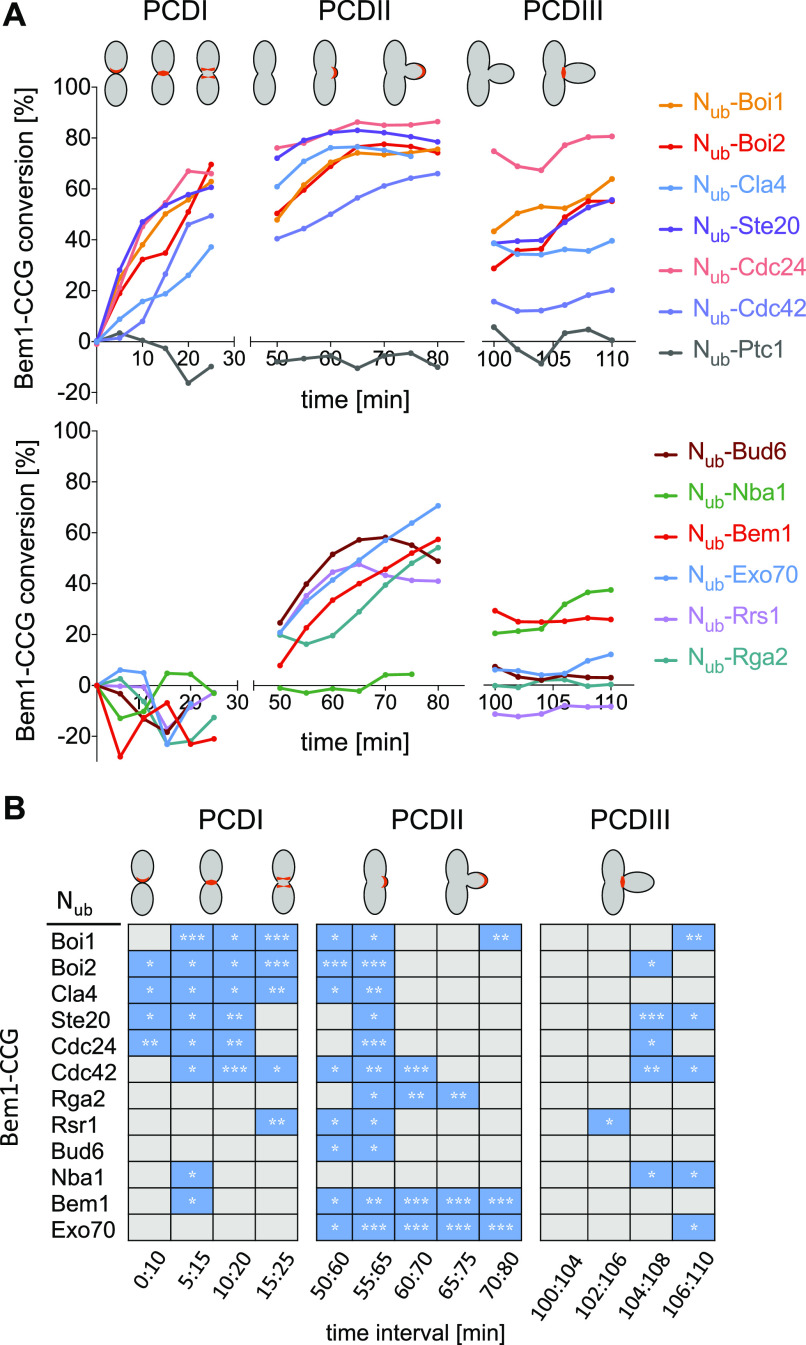
SPLIFF analysis of yeast zygotes formed by the fusion of a-cells expressing Bem1CCG and α-cells expressing the indicated N_ub_-fusions. **(A)** Plotted are the conversions of Bem1CCG to Bem1CC (%) over time. GFP- and mCherry fluorescence intensities were measured at sites of polarized Bem1 locations as indicated in red in the cartoons of the upper panel. Shown are fitted lines calculated from single-cell experiments. **(B)** Interaction profiles for Bem1. Blue boxes indicate a significant increase in conversion over the indicated time. For values below 70% of conversion, significant slopes have to be 1 (% conversion/min) or larger. For values above 70%, slopes have to be positive and significant. To be counted as interaction, the criteria must not be met by the negative control in the respective time window (*** = *P*-value < 0.001; ** = *P*-value < 0.01; * = *P*-value < 0.05).

**Figure S5.**
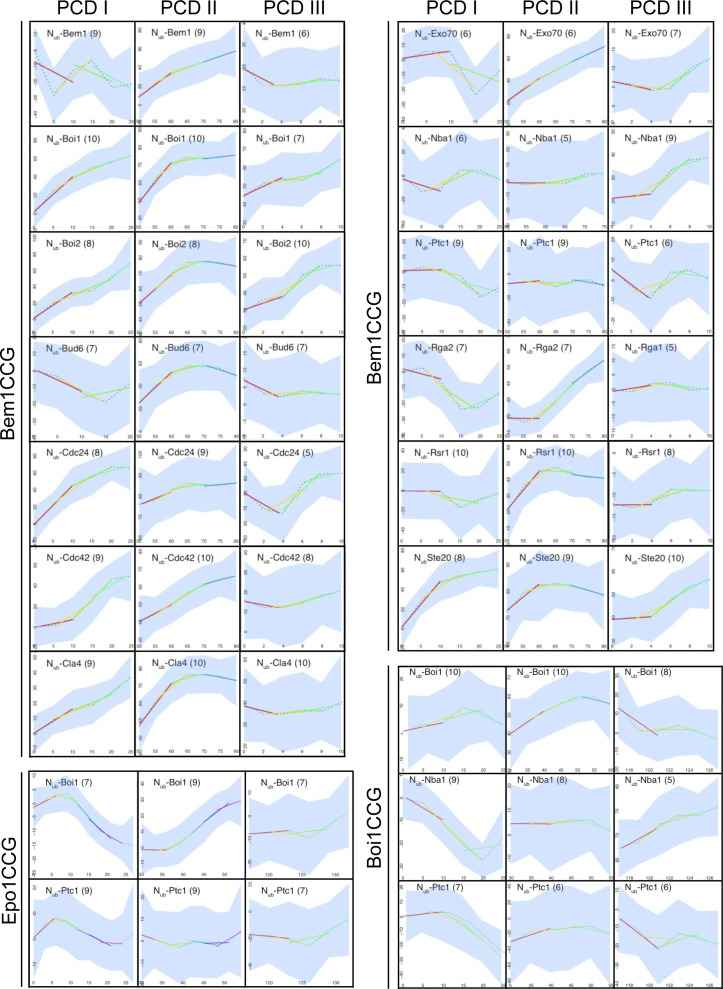
Calculated slopes of the conversions of Bem1-, Boi1-, and Epo1CCG during PCDI, PCDII, and PCDIII. Data are derived from Table S1. Dotted blue lines represent the nonparametric local regression (loess) fitted lines, whereas the corresponding confidence intervals (95%) are shown in light blue. Generalized additive model fitted over two time window intervals are represented by the colored segments. Numbers of cells are indicated in parentheses.

A Bem1-mCherry-Cub-RGFP fusion protein (Bem1CCG) was expressed from its genomic locus under the control of the conditional *MET17* promoter in *MATa* cells. All N_ub_ fusions were expressed in α-cells from their native promoters, except N_ub_-Cdc42 and N_ub_-Exo70 that were under control of the non-induced P_*CUP1*_ promoter. N_ub_-Rsr1 was included in the analysis as it generated under its native expression levels a strong interaction signal with Bem1CRU (Fig S4F). Mating and fusion of the a- and α cells allowed Bem1CCG and the respective N_ub_ fusion to interact. Green and red fluorescence were then measured during one cell cycle at the site of cell fusion (PCDI), at the cell front during bud site assembly and bud growth (PCDII), and finally at the bud neck from completion of acto-myosin ring contraction until cell abscission (PCDIII) (Fig S4G). The ratios of the fluorescence intensities (IFs) from individual single-cell experiments were fitted into a single curve and plotted as percentage of N_ub_-induced conversion of Bem1CCG to Bem1CC against time after cell fusion (Fig 8 and Table S1). A regression-based significant positive slope over two time intervals was taken as evidence for interaction (Figs 8B and S5 and Table S2). No significant increase or a decrease in the relative amount of conversion was considered as absence of interaction. It is important to remind that the absence of an interaction signal indicates “no interaction” only within the detection limit of SPLIFF. The interaction partners of Bem1 fall into the following categories: Ste20, Cdc24, Rsr1, Boi1, Boi2, and Cdc42 interacted with Bem1 during all three phases (Figs 8A and B and S5 and Tables S1 and S2). Cla4 interacted with Bem1 only during PCDI and II. Bud6 interacted with Bem1 only during bud formation and growth (PCDII). Nba1 interacted with Bem1 shortly during PCDI and throughout cytokinesis (PCDIII), whereas Exo70 interacted during PCDII, and in PCDIII, only shortly before cell separation (Figs 8A and B and S5 and Table S2). The multimerization of Bem1 could be observed during a single time frame in PCDI and throughout PCDII. We can, further, differentiate between Bem1 interactions that last through the entire PCDII (Bem1, Boi1, Exo70, Cdc42, and Cdc24) and those that are detectable in small buds only (Bud6 and Rsr1) (Figs 8A and B and S5 and Table S2). The interaction between Bem1 and Rga2 stalled during bud formation and picked up after 10 min into bud growth to continue as long as Bem1 remained at the cortex (Figs 8 and S5 and Tables S1 and S2). The interaction signals between Bem1 and Boi1, Boi2, Cla4, and Ste20 reached a plateau after ∼20 min into bud growth. The slight increase of conversion was considered as sign of a continuing yet diminished interaction between Bem1 and Boi1 during the remaining phase of bud growth (Figs 8A and B and S5 and Table S2). The decrease in the ratio of converted Bem1CCG in the N_ub_-Ste20–, N_ub_-Cla4–, and N_ub_-Boi2–expressing cells might already indicate a loss of interaction between Bem1 and the N_ub_ fusions during the transition from bud growth to mitosis. However, it has to be noted that conversion ratios at or above 80% are very difficult to interpret and that no increase or a slight decrease do not necessarily have to reflect the absence of interaction. During cytokinesis, Boi1/2, Ste20, Rsr1, Cdc24, Cdc42, Exo70, and Nba1 were detectably associated with Bem1 (Figs 8 and S5 and Tables S1 and S2), whereas N_ub_-Cla4, N_ub_-Bud6, N_ub_-Rga2, and N_ub_-Bem1 did not convert Bem1CCG during abscission (PCDIII, Figs 8A and B and S5 and Tables S1 and S2).

Table S1 Calculated ratios (FD) of CCG- to CC conversion in percent.

Table S2 Slopes of the calculated curves and their significance.

### SH3_Boi1_ switches between interaction partners during the cell cycle

The detection of the Bem1–Bem1 interaction requires the oligomerization of the Boi proteins, whereas the proximity between Bem1 and Nba1 is mediated by the simultaneous interactions of both proteins with Boi1/2 (Figs 2 and 3).

**Figure 9.**
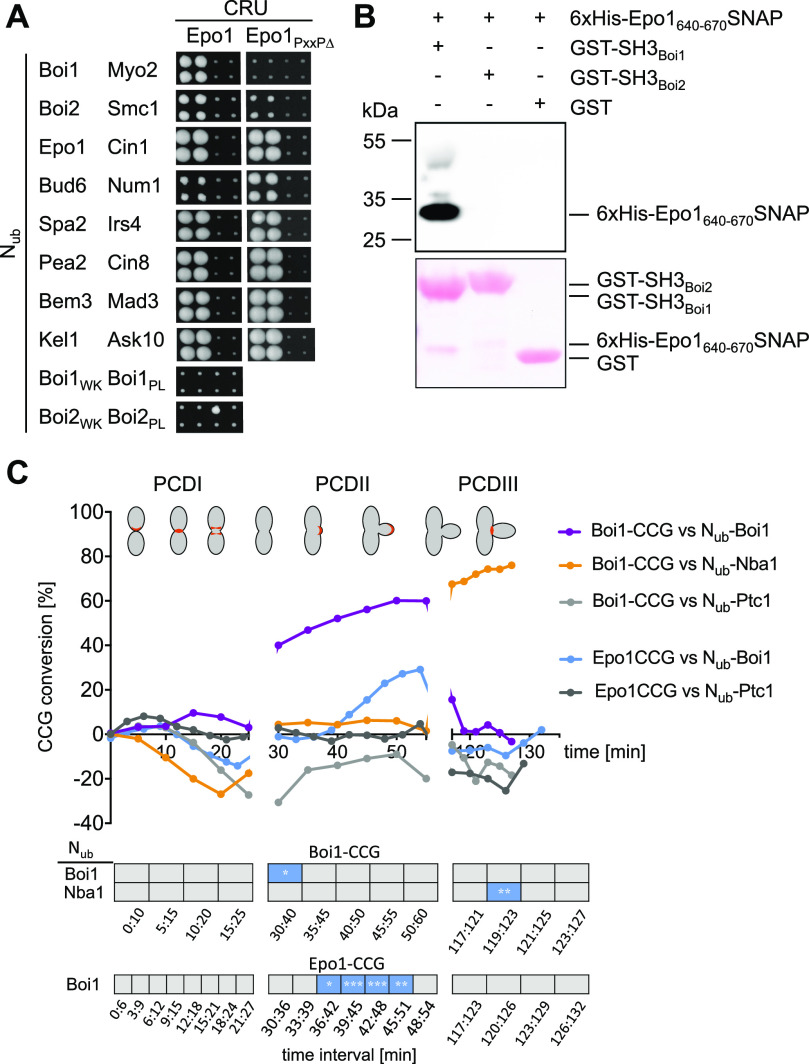
Epo1 interacts with Boi1 during budding. **(A)** As in Fig 1A but with cells expressing Epo1CRU or Epo1_654-661∆_ (Epo1_*PxxP*∆_) CRU with the indicated N_ub_ fusions. **(B)** Extracts containing a 6xHIS SNAP-tag fusion to Epo1_640-670_ were incubated with GST-Boi1-, GST-Boi2-, or GST-coupled beads. Glutathione-eluates were stained with Ponceau (lower panel), and anti-His antibodies (upper panel) after SDS–PAGE and transfer onto nitrocellulose. **(C)** SPLIFF analysis: a-cells expressing Epo1CCG or Boi1CCG were mated with α-cells expressing the indicated N_ub_ fusions. Upper panel: The measured fluorescence intensities were processed and visualized as in Fig 8A. Significance of slopes of fitted lines across time intervals are shown in the lower panel (*** = *P*-value < 0.001; ** = *P*-value < 0.01; * = *P*-value < 0.05).

We tested the consistency of our SPLIFF analysis by measuring Boi1CCG against N_ub_-Boi1 and N_ub_-Nba1. In agreement with the time dependency of Bem1 multimerization and the formation of the Nba1–Bem1 complex, Boi1CCG was converted to Boi1CC by N_ub_-Boi1 during bud growth and not during abscission, whereas Boi1CCG was converted by N_ub_-Nba1 only during abscission (Figs 8B and 9C and Tables S1 and S2). Boi1CCG/N_ub_-Nba1, thus, lacked the interaction signal observed between Bem1CCG and N_ub_-Nba1 during PCDI. The reported Boi1/2–independent interaction between Bem1 and Nba1 might account for this apparent discrepancy (Fig 8) (Meitinger et al, 2014). The high level of Boi1CCG conversion at the beginning of PCDIII might indicate that Boi1/2-Bem1 arrive together with Nba1 as a preformed complex at the bud neck. The time resolution of our assay cannot distinguish this scenario from our preferred interpretation that conversion occurred exclusively at the neck.

Besides binding to Nba1 and Fir1, SH3_Boi1/2_ interact with additional proteins and might expand the influence of the Bem1–Cdc24 complex to further processes (Tonikian et al, 2009; Kustermann et al, 2017). To test whether other SH3_Boi_ interactions are also cell cycle–specific, we investigated the interaction between Epo1 and Boi1. Epo1 links the cortical endoplasmic reticulum to the polarisome and was shown to bind Boi1/2 (Neller et al, 2015). Mutations that inactivate SH3_Boi1_ or a mutation of the predicted Boi1-binding motif in Epo1 abolished the interaction between both proteins (Fig 9A). A pull down of this binding motif with a GST fusion to SH3_Boi1_ proved its direct interaction (Fig 9B). Epo1 and Nba1 thus compete for the same binding site in Boi1. SPLIFF picked up the interaction between N_ub_-Boi1 and Epo1CCG for the first time during bud growth (Figs 9C and S5 and Tables S1 and S2). No interaction could be recorded for Epo1 during PCDIII at the bud neck (Fig 9C). Although sharing the same interaction site, Epo1 and Nba1 interacted with Boi1–Bem1 at different stages of the cell cycle.

## Discussion

Bem1 is a central scaffold protein for the Cdc42 pathway that is essential in some but not all *Saccharomyces cerevisiae* strains (Dowell et al, 2010). Eliminating the GAP activity of Bem3 and thus increasing the concentration of active Cdc42 at the cortex especially during bud formation rescues the otherwise lethal deletion of *BEM1* in the strain JD47, whereas the overexpression of a Cdc42_GTP_- and membrane-binding fragment of Gic2 counteracts the positive effect of the *BEM3* deletion (Knaus et al, 2007). Whether yeast cells of a certain strain can live without Bem1 thus seems to depend on the remaining concentration of active Cdc42 at the cortex. A central fragment of Bem1 harboring the SH3_b_ domain with its neighboring Cdc42_GTP_ binding element and a C-terminal fragment, containing the PB1 and PX domain, independently rescue a *bem1∆* strain. The C-terminal fragment binds strongly to Cdc24 but does not connect Cdc24 to Cdc42 effector proteins or to the cortex. We propose, in line with published data, that the C-terminal fragment increases the concentration of Cdc42_GTP_ by stimulating the activity of Cdc24 (Shimada et al, 2004; Smith et al, 2013; Rapali et al, 2017). The central SH3_b_CI fragment interacts with active Cdc42 and four Cdc42 effectors and requires both of its binding sites to rescue *bem1∆* cells. Our genetic analysis and published data suggest that the functional units SH3_b_, CI, and PB1 of Bem1 co-operate and form a chain of binding sites that funnel active Cdc42 from its source to its targets (Kozubowski et al, 2008).

Ste20 and Cla4 are the essential ligands of SH3_b_CI under limiting concentrations of Cdc42_GTP_ (Fig 5A). How can the isolated SH3bCI without connection to Cdc24 still stimulate the PAKs? A comparison between the binding characteristics of the non-essential Boi1/2-PxxP sites and the essential Ste20/Cla4-PxxP sites suggests a molecular mechanism. Bem1 and all its SH3b ligands are concentrated at the cell tip during bud formation and growth. The cortex localizations of the PAKs but not of Boi1/2 strictly depend on Cdc42_GTP_ (Peter et al, 1996; Leberer et al, 1997; Wild et al, 2004; Kustermann et al, 2017). The isolated binding motifs of Cla4 and of Boi1/2 bind with similar affinities to SH3_b_, whereas the SH3_b_-binding motif of Ste20 displays a significantly higher in vitro affinity (Gorelik & Davidson, 2012). SPLIFF analysis shows that both PAKs and Boi1/2 interact with Bem1 during bud formation and growth. Nevertheless, and in contrast to Boi1/2, both PAKs do not measurably contribute to the cortical localization of SH3_b_CI or of full-length Bem1. We hypothesize that Bem1 interacts stronger with the inactive PAKs than with their cortex-localized Cdc42_GTP_-bound forms. To explain the stimulatory activity of SH3_b_CI, we postulate that SH3_b_CI might open the CRIB domains of the PAKs to actively load them with the CI-attached Cdc42_GTP_ (Lamson et al, 2002). The Cdc42_GTP_-bound CRIB domain might then release the auto-inhibition of the kinases and at the same time, impair the interaction with SH3_b_. This sequence describes Bem1 not as a passive scaffold but more similar to the kinase scaffold Ste5 as a coactivator that regulates through binding the activity of the PAKs and stimulates the synthesis and the transfer of Cdc42 (Bhattacharyya et al, 2006). Support for our model comes from two observations: 1. Bem1 needs the fully functional SH3bCI to activate Ste20 and Cla4 also during osmostress (Chang et al, 1999; Tanaka et al, 2014). 2. Scd2, the Bem1 homolog from *Schizosaccharomyces pombe*, binds with its second SH3 domain the Ste20 homologs Shk1 and thereby increases the auto-phosphorylation activity of the kinase (Chang et al, 1999; Tanaka et al, 2014).

**Figure 10.**
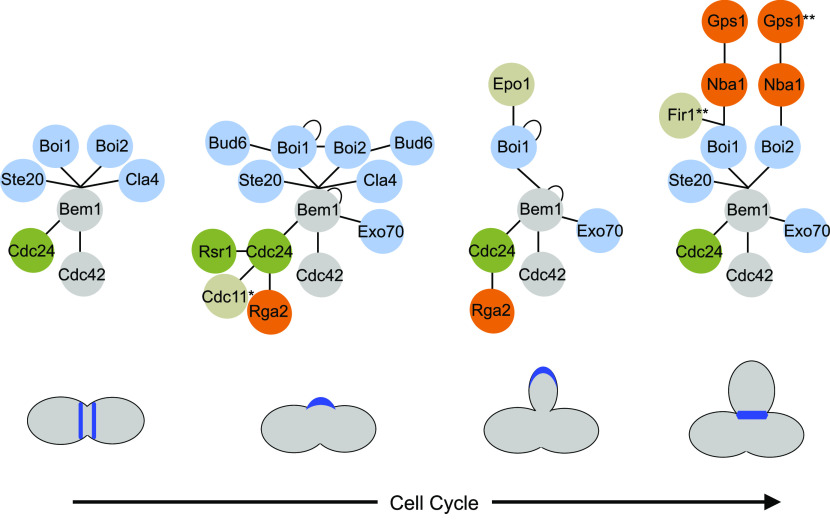
Interaction networks of Bem1 during PCDI (10–20 min), early and late PCDII (50–55 min, 60–80 min), and PCDIII (104–110 min). Green color indicates proteins that promote, orange color indicates proteins that reduce active Cdc42. Blue color indicates effectors of Cdc42 or proteins that bind to effectors (Bud6-Bni1). Epo1 binds to the same site of Boi1 as Nba1 or Fir1 but at a different cell cycle phase. *The time point of interaction between Bem1-Cdc24-Cdc11 was obtained from a previous study (Chollet et al, 2020). **The time point of Fir1- and Gps1 binding to Bem1 were indirectly derived through their effects on Bem1 localization (Fig 7).

Localizing Cdc24, stimulating its activity, and channeling Cdc42 to its effectors are separable activities of Bem1 that cooperate in the full-length protein to generate and read the gradient of active Cdc42 (Smith et al, 2013; Woods et al, 2015; Rapali et al, 2017). A temporal map of the interaction network of this complex might thus reveal where at a given time the activated Cdc42 is preferentially directed. SPLIFF is one of the very few techniques that allow analysis of temporal aspects of protein–protein interactions in living cells. The cleavage of GFP from the mCherry-C_ub_-GFP coupled interaction partner gives a robust ratiometric output for interaction. However, to define the time point of interaction, one has to record the change of this value over time in single cells. The closer the chosen time points, the smaller the change and the less sensitive the measurement. To prove the significance of the observation, measurements have to be repeated in different cells at comparable positions of the cell cycle. In addition, the depletion of the uncleaved mCherry-C_ub_-GFP fusion upon interaction reduces the maximal response during the later time points of the measurements. These features limit the sensitivity of the assay and sometimes blur the distinction between no or rarely occurring interactions. Despite these shortcomings, the application of SPLIFF provided a unique temporal interaction profile of the scaffold protein Bem1. As Bem1 links active Cdc42 to its effectors, Fig 10 summarizes the cellular flow of Cdc42_GTP_ through the cell cycle. During bud site formation and bud growth, Cdc42 is channeled directly to Exo70 and possibly from Boi1/2 to the other Cdc42-activated exocyst component Sec3. Boi1/2 were shown to recruit Bud6 and Bni1 to sites of active exocytosis (Glomb et al, 2020). The temporal formation of the Bem1–Boi1/2–Bud6 complex might thus boost the Bni1-catalyzed actin filament formation and organization during bud site assembly and in small buds (Adams et al, 1990; Dong et al, 2003; Glomb et al, 2020). As the binding sites of Bem1 for Boi1/2 and Exo70 do not overlap, a super-complex that stimulates and coordinates actin assembly and vesicle fusion during bud assembly and early growth seems plausible (Adamo et al, 2001; Liu & Novick, 2014; Kustermann et al, 2017; Glomb et al, 2020). This complex disassembles in large buds and does not detectably form during cytokinesis (Figs 8 and 10).

The PAKs Ste20 and Cla4 contact Bem1 at the same site as Boi1/2 and form alternative, exclusive interaction states (Fig 10). The Cla4-Bem1-Cdc42-Cdc24 and Ste20-Bem1-Cdc42-Cdc24 interaction states coexist with the Boi1/2-Bem1-Cdc42-Cdc24 interaction states except during abscission where only Ste20-Bem1-Cdc42-Cdc24 and Boi1/Boi2-Bem1-Cdc42-Cdc24 are detectable (Figs 8 and 10). The persistent activation of Ste20 correlates with its role during cytokinesis and its early, CDK-independent activation in the next cell cycle (Moran et al, 2019).

The Boi1/2–Bem1–Cdc24 complex is anchored by Nba1 and additionally by Fir1 to the bud neck (Fig 7F). Fir1 is known to delay cell separation by inhibiting the cell separation kinase Cbk1 (Brace et al, 2019). *NBA1* is synthetic lethal to a certain allele of the essential cytokinesis factor IQGAP (in yeast: *IQG1*), suggesting that Nba1 stimulates abscission (Tian et al, 2014). A better understanding of the functions of Nba1 and Fir1 might reveal how the Bem1/Cdc24 complex moderates cytokinesis.

The PAKs perform many other functions besides their roles during cytokinesis and bud formation (Drogen et al, 2000; Hofken & Schiebel, 2002; Tanaka et al, 2014). The general Cdc42 sensitivity of cells lacking the Bem1-binding sites in *STE20* and *CLA4* indicates that the PAK-Bem1-Cdc42-Cdc24 interaction states are the operative units for many if not all PAK activities.

## Materials and Methods

### Growth conditions and cultivation of yeast strains

All yeast strains were derivatives of JD47, a descendant from a cross of the strains YPH500 and BBY45 (Sikorski & Hieter, 1989; Bartel et al, 1990; Madura et al, 1993; Dohmen et al, 1995). Cultivation of yeast was performed in standard SD or YPD media at 30°C or the indicated temperatures as described (Dünkler et al, 2012). Media for split-ubiquitin interaction assay and selection for the loss of centromeric *URA3*-containing plasmids comprised 1 mg/ml 5-fluoro-orotic acid (5-FOA; Formedium).

### Construction of plasmids, gene fusions, and manipulations

Construction of N_ub_ and C_ub_ gene fusions as well as GFP-, mCherry-, or mCherry-C_ub_-RGFP (CCG) fusions was performed as described (Wittke et al, 1999; Dünkler et al, 2012; Moreno et al, 2013; Neller et al, 2015). Bem1CRU/-GFP/-CCG were constructed by genomic in-frame insertions of the *GFP*-, *CRU*-, or *CCG* modules behind the coding sequences of *BEM1* or its alleles. In brief, a PCR fragment of the C-terminal region of the respective target gene lacking the stop codon was cloned via *Eag*I and *Sal*I restriction sites in front of the CRU-, GFP-, mCherry-, or CCG-module on a pRS303, pRS304, or pRS306 vector (Sikorski & Hieter, 1989). Plasmids were linearized using unique restriction sites within this sequence and transformed into yeast cells for integration into the genomic target ORF. Colony PCR with diagnostic primer combinations was used to verify the successful genomic integration. Centromeric plasmids expressing different fragments of *BEM1* were obtained by ligation of PCR fragments spanning the respective region of *BEM1* behind the sequence of the *P*_*MET17*_-GFP module on the pRS313 vector (Table S3) (Sikorski & Hieter, 1989). Mutations in the coding region of *BEM1*, *STE20*, *CLA4*, or *BOI1* were obtained by overlap-extension PCR using plasmids containing the corresponding ORFs as templates.

Table S3 List of plamsids.

Insertion of mutations into the *BEM1*, *STE20*, *BOI1*, or *CLA4* loci were performed in yeast strains lacking the ORFs of the respective genes but still containing their 5′ and 3′ UTR sequences. Mutations were introduced in the respective genes on an integrative pRS vector containing the up- and downstream sequences of the gene (Sikorski & Hieter, 1989). Yeast strains lacking the corresponding ORF were then transformed with the mutated gene on the integrative vector linearized in the promoter sequence of the gene. Successful integration was verified by diagnostic PCR.

Alternatively, insertion of genomic mutations was achieved by CRISPR/Cas9 (Laughery et al, 2015). To introduce the mutations at the chosen sites, guideRNA sequences were cloned into pML plasmids and co-transformed with oligonucleotides harboring the desired mutations. Successful manipulations were verified by PCR product sequencing of the respective genomic ORFs. Details of the introduced mutations are listed in Table S4.

Table S4 List of yeast strains.

In certain strains, the native promoter sequence was replaced by *P*_*MET17*_ through recombination with a PCR fragment generated from pYM-N35 and primers containing sequences identical to the respective genomic locations at their 5′ ends (Janke et al, 2004). GST fusions were obtained by placing the ORF of the respective gene or gene fragment in frame behind the *Escherichia coli GST* sequence on the pGEX-2T plasmid (GE Healthcare) using *Bam*HI and *Eco*RI restriction sites. Fusions to the human O6-Alkyl-DNA transferase (SNAP-tag; New England Biolabs) were expressed from plasmid pAGT-Xpress, a pET-15b derivative (Schneider et al, 2013). Gene fragments were inserted in frame into a multi-cloning site located between the upstream *6xHIS*-tag–coding sequence and the downstream *SNAP*-tag–coding sequence. The *6xHIS*-tag fusions were obtained by placing the ORF of the respective gene or gene fragment behind the *E. coli 6xHIS*-tag sequence on the previously constructed pAC plasmid (Schneider et al, 2013).

Gene deletions were performed by one step PCR-based homologous recombination using pFA6a natNT2, pFA6a hphNT1, pFA6a kanMX6, pFA6a CmLEU2, and pFA6a HISMX6 as templates (Bähler et al, 1998; Longtine et al, 1998; Janke et al, 2004; Schaub et al, 2006). Lists of plasmids and yeast strains used in this study can be found in Tables S3 and S4 of the supplemental information. Plasmid maps can be obtained upon request.

### Split-Ub interaction analysis

Bem1 was fused to the C-terminal half of Ubiquitin followed by Ura3 carrying an arginine at its N terminus to create Bem1-C_ub_-RUra3 (Bem1CRU). Upon co-expression of a binding partner of Bem1 carrying the N-terminal half of Ub (N_ub_-X) at its N terminus, N_ub_ and C_ub_ are brought into close proximity and the native-like Ubiquitin is reconstituted from its two halves. Ub-specific proteases cleave off the RUra3 module from C_ub_ and the exposed arginine initiates the degradation of RUra3. As a consequence, cells have no Ura3 activity and do not convert 5-FOA in the toxic 5-fluoro-uracil. The growth of cells on media containing 5-FOA is thus taken as evidence of interaction between the C_ub_- and N_ub_-coupled fusion protein (Johnsson & Varshavsky, 1994; Wittke et al, 1999).

#### Array analysis

A library of 548 different α-strains each expressing a different N_ub_ fusion were mated with a *B*EM*1*-*C*_*ub*_*-R-URA3* (Bem1CRU), *B*EM*1*_*WK*_-*C*_*ub*_*-R-URA3* (Bem1_WK_CRU), or *B*EM*1*_*PB1*∆_-*C*_*ub*_*-R-URA3* (Bem1_PB1∆_CRU) expressing a-strain. Diploids were transferred as independent quadruplets on SD media containing 1 mg/ml 5-FOA. Expressions of the N_ub_ fusions were under control of the copper inducible *P*_*CUP1*_-promoter. Media contained different concentrations of copper to adjust the amount of the N_ub_ fusions (Dünkler et al, 2012).

#### Individual Split-Ub interaction analysis

CRU and N_ub_ expressing strains were mated or co-expressed in haploid cells and spotted onto the medium containing 1 mg/ml FOA and different concentrations of copper in four 10-fold serial dilutions starting from OD_600_ = 1. Growth at 30°C was recorded every day for 3–5 d.

### Complementation analysis

*bem1∆* cells expressing *BEM1* from a *URA3*-containing centromeric vector and fragments of *BEM1* from an *HIS3*-containing vector were either streaked directly or spotted as 10-fold serial dilutions onto SD His^−^ media containing 1 mg/ml 5-FOA. As 5-FOA is converted by Ura3 to the toxic 5-fluoro-uracil, the medium counter-selects against the presence of the *BEM1*-expressing *URA3* vector.

### Mating efficiency

Saturated cultures of JD47 cells containing the respective allele of *STE20* and expressing Gic2_PBD_ from a centromeric plasmid under the control of a *P*_*MET17*_ promoter and JD53 cells carrying a Kanamycin-tagged *PTC1* gene and expressing Gic2_PBD_ were resuspended in media containing no or 70 μm methionine and grown for 6 h at 30°C. Cells were adjusted to an OD_600_ of 1 and equal amounts of JD53 and JD47 cells mixed and incubated for 4 h at 30°C. Cells were diluted 1/20 and 250 μl of each mating were spread on media selecting for diploids. Colony numbers were counted after 2 d at 30°C.

### Preparation of yeast cell extracts

Exponentially grown yeast cell cultures were pelleted and resuspended in yeast extraction buffer (50 mM Hepes, 150 mM NaCl, and 1 mM EDTA) with 1× protease inhibitor cocktail (Roche Diagnostics). Cells were lysed by vortexing them together with glass beads (threefold amount of glass beads and extraction buffer to pellet weight) 12 times for 1 min interrupted by short incubations on ice. The obtained yeast cell extracts were clarified by centrifugation at 16,000*g* for 20 min at 4°C.

### Recombinant protein expression and purification from *E. coli*

All proteins were expressed in *E. coli* BL21DE3 cells. GST-Bud6_1-320_ was expressed at 30°C for 5 h in LB medium after induction with 1 mM IPTG. GST fusions to SH3 domains of Boi1 and Boi2 and 6xHis-Nba1_202-289_-SNAP were expressed at 18°C in the SB medium for 20 h after induction with 0.1 mM IPTG. Cells were pelleted, washed once with PBS, and stored at −80°C until lysis. All subsequent purifications were carried out on an Äkta Purifier chromatography device (GE Healthcare). Cells expressing GST fusion proteins were resuspended in PBS containing protease inhibitor cocktail (Roche Diagnostics) and lysed by lysozyme treatment (1 mg/ml, 30 min on ice), followed by sonication with a Bandelin Sonopuls HD 2070 (Reichmann Industries service). Extracts were clarified by centrifugation at 40,000*g* for 10 min at 4°C, and the proteins were purified using a 5-ml GSTrap column (GE Healthcare) and subsequent size exclusion chromatography on a Superdex 200 16/60 column versus HBSEP buffer (10 mM Hepes, 150 mM NaCl, 3 mM EDTA, and 0.05% Tween 20, pH 7.4). Purified protein was concentrated and stored on ice.

6xHis-Nba1_202-289_-SNAP–expressing cells were lysed in IMAC buffer (50 mM KH_2_PO_4_, 300 mM NaCl, and 20 mM imidazole containing protease inhibitor cocktail) as described above, and enriched protein was obtained by imidazole gradient elution from a 5-ml HisTrap HP column (GE Healthcare), followed by size-exclusion chromatography.

### GST pull-down assay

GST-tagged proteins were immobilized on Glutathione Sepharose beads (GE Healthcare) directly from *E. coli* extracts. After 1-h incubation at 4°C with either yeast extracts or purified proteins under rotation at 4°C, the beads were washed three times with the respective buffer. Bound material was eluted with GST elution buffer (50 mM Tris and 20 mM reduced glutathione) and analyzed by SDS–PAGE followed by Coomassie staining and immunoblotting with anti-His (dilution: 1:5,000; Sigma Aldrich), or anti–GFP-antibodies (dilution 1:1,000; Roche Diagnostics).

### Surface plasmon resonance measurements

Binding affinities were measured using purified and immobilized GST-SH3_Boi1_ or GST-SH3_Boi2_ as ligands on an anti-GST chip on a Biacore X100 device (GE Healthcare). HBSEP buffer was used as the running buffer in all experiments. The chip was prepared by covalent coupling of an anti-GST antibody (GE Healthcare) as capture molecule to the dextran surface of both flow cells of a CM5 chip (GE Healthcare) using an amine coupling kit (GE Healthcare). GST-tagged ligand proteins were captured on the detection flow cell of the chip, and free GST was captured on the reference flow cell. Purified 6xHis-NBA1_202-289_-SNAP as an analyte molecule was prepared in suitable concentrations in running buffer and kinetics were measured with constant flow over the previously prepared chip. Regeneration after each cycle was achieved by a 20-s injection pulse with 10 mM glycine, pH 2.0. The equilibrium binding constant K_D_ was subsequently determined by the X100 evaluation software using background subtracted sensograms. All measurements were performed at least as triplicate.

### Fluorescence microscopy

For microscopic inspection, yeast cells were grown overnight in SD medium, diluted 1:8 in 3–4 ml fresh SD medium, and grown for 3–6 h at 30°C to mid-log phase. About 1 ml culture was spun down, and the cell pellet resuspended in 20–50-μl residual medium. 3 μl was spotted onto a microscope slide, and the cells were immobilized with a coverslip and inspected under the microscope. For time-resolved imaging, 3 μl of prepared cell suspension was mounted on an SD-agarose pad (1.7% agarose), embedded in a customized glass slide, and sealed by a coverslip fixed by parafilm stripes. Imaging was started after 15–30 min recovery at 30°C. SPLIFF and other time-lapse experiments were observed under a wide-field fluorescence microscope system (DeltaVision; GE Healthcare) provided with a Olympus IX71 microscope, a steady-state heating chamber, a CoolSNAP HQ2 and CascadeII512-CCD camera both by Photometrics, a U Plan S Apochromat 100 Å ∼ 1.4 NA oil ∞/0.17/FN26.5 objective and a Photofluor LM-75 halogen lamp. Images were visualized using softWoRx software (GE Healthcare) and adapted z series at 30°C. Exposure time was adapted to the intensity of GFP and mCherry signal for every fluorescently labeled protein to reduce bleaching and phototoxicity. Further analyses used an Axio Observer spinning-disk confocal microscope (Zeiss), equipped with an Evolve512 EMCCD camera (Photometrics), a Plan-Apochromat 63 Å∼/1.4 oil DIC objective, and 488- and 561-nm diode lasers (Zeiss). Images were analyzed with the ZEN2 software (Zeiss).

### Quantitative analysis of microscopy data and SPLIFF measurements

Microscopy data were processed and analyzed using ImageJ64 1.49 software. For standard fluorescence signal quantification, three regions of interest (ROIs) were determined, first the signal of interest (e.g., tip, bud neck), second a region in the cytosol, and third a randomly chosen position outside of the cell (background). The mean gray values of each ROI (*I*_*fluorescence*_, *I*_*cytosol*_, and *I*_*background*_) were quantified after z-projection. To compare the fluorescence signals of a protein in certain strains, the relative fluorescence (*I*_*relative*_) signal of the protein was calculated after subtraction of the background.

$$I_{\mathrm{relative}}\;=\;I_{\mathrm{fluorescence}}\;-\;I_{\mathrm{background}}\text{/}I_{\mathrm{cytosol}}\;-\;I_{\mathrm{background}}$$

### SPLIFF analysis

SPLIFF analysis for temporal and spatial characteristics of Bem1-, Boi1-, and Epo1-CCG interactions was performed as described (Moreno et al, 2013; Dunkler et al, 2015). a-cells expressing the P_*MET17*_ promoter–controlled CCG fusions were grown in SD medium without methionine and mixed with *MAT* α cells expressing N_ub_*-HA* fusions either under their native or the P_*CUP1*_-promoter. After mixing, the cells were immobilized on an SD agarose pad and mating-induced interaction was monitored by three channel z-stack (5 × 0.6 μm) microscopy every 2, 3, or 5 min. z-slices with fluorescence signals were projected by SUM projection. The FI of mCherry and GFP channels were determined by integrated density measurements of the ROI and a region within the cytosol. For each time point and channel, the intracellular background was subtracted from the localized signal to obtain the localized fluorescence intensity (FI_red_ and FI_green_). The values were normalized to the time point before cell fusion. The resulting relative fluorescence intensity RFI(t) was then used to calculate the conversion FD(t):

$$\mathrm{FD}(t)\;=\;\mathrm{RF}I_{\mathrm{red}}\;-\;\mathrm{RF}I_{\mathrm{green}}\text{/}\mathrm{RF}I_{\mathrm{red}}$$

FD(t) as a readout of CCG- to CC conversion describes its temporal progress in percent. Excel was used for initial calculations.

### Regression and slope estimation of SPLIFF analysis

To test significance of increment of percent conversion over time, the non-parametric local regression (loess) (R Core Team, 2019) was first fitted by taking all biological replicates across the entire time window. The fitted line was then used for the generalized additive model (Hastie, 2019). For the generalized additive model, two time intervals with one time point sliding window were used to estimate the slope of rate of change of percent of conversion. Positive slopes with a *P*-value cutoff of 0.05 were considered as statistically significant.

### FRAP experiments

FRAP experiments were performed as described elsewhere with an iMIC digital microscope with a 60× objective (Till Photonics) at RT (Phair et al, 2004). Pictures were acquired with a series of five z-slices each separated by 0.5 μM. Four images were taken before the ROI was bleached with 100% laser power, a dwell time of 1.2 s/μm^2^, a line overlap of 42%, and an experimental loop count of 10–20. Pictures were taken at a constant time interval of 0.9 s after bleaching except SH3_Boi1_-GFP where signal recovery was measured each 0.25 s in a single z-layer. Initial z-slice projection and fluorescence quantification was performed with the software iMIC Offline analysis. Alternatively, an Axio Observer spinning-disc confocal microscope (Zeiss), equipped with a Zeiss Plan-Apochromat 63 Å∼/1.4 oil DIC objective, a 488-nm diode lasers, and an UGA-42 photo-manipulation system was used (Rapp OptoElectronic). Initial signal measurements were ImageJ based. Subsequently, all data sets were double-normalized using Excel. The software Prism 6.0 (GraphPad) was used for the fitting of the double-normalized data to a one-phase association curve.

### Statistical evaluation

GraphPad Prism was applied for statistical data evaluation. The distributions of the data sets were analyzed by the D’Agostino and Pearson normality test. *t* tests were used to analyze data following a normal distribution, whereas Mann–Whitney U tests were used for data that did not pass these criteria. The one-way ANOVA or the Kruskal–Wallis ANOVA tests were used to compare data sets from more than two groups.

## Supplementary Material

Reviewer comments
